# Hyperoside alleviates endometrial stromal cell senescence in unexplained recurrent spontaneous abortion via DHX9‐mediated R‐loop resolution

**DOI:** 10.1002/imt2.70150

**Published:** 2026-07-13

**Authors:** Yuepeng Jiang, Hongli Zhao, Xinyi Ding, Haoling Zhang, Yadong Guo, Yiming Ma, Lingyi Cai, Qingnan Fan, Ruisi Peng, Fuyuan Yang, Doblin Sandai, Xianling Cao, Jiali Yao, Wenyi Wang, Zhiheng Lin, Wangzheqi Zhang, Jialin He, Aihua Zhang, Xiaoxuan Zhao

**Affiliations:** ^1^ Department of Traditional Chinese Medicine (TCM) Gynecology Hangzhou TCM Hospital Affiliated to Zhejiang Chinese Medical University Hangzhou China; ^2^ College of Basic Medical Sciences Zhejiang Chinese Medical University Hangzhou China; ^3^ Department of Biomedical Sciences, Pusat Kanser Tun Abdullah Ahmad Badawi University Sains Malaysia, Kepala Batas Pulau Pinang Malaysia; ^4^ Shanghai Tenth People's Hospital Tongji University Shanghai China; ^5^ Faculty of Chinese Medicine Macau University of Science and Technology Macau China; ^6^ The Third Clinical Medical College Zhejiang Chinese Medical University Hangzhou China; ^7^ Department of Medicine Harvard Medical School (HMS), Joslin Diabetes Center Harvard University Boston Massachusetts USA; ^8^ Clinical Cancer Institute, Center for Translational Medicine Naval Medical University Shanghai China; ^9^ Juntendo University Tokyo Japan; ^10^ Department of Gynecology, Longhua Hospital Shanghai University of Traditional Chinese Medicine Shanghai China; ^11^ Naval Medical University Shanghai China; ^12^ Hainan Medical University Haikou China

**Keywords:** cell senescence, cGAS‐STING signaling, decidualization, hyperoside, R‐loop, unexplained recurrent spontaneous abortion

## Abstract

Decidualization deficiency is a hallmark pathology of unexplained recurrent spontaneous abortion (URSA), but the undefined molecular drivers hinder the development of effective therapies. Hyperoside, a bioactive flavonoid from Hypericum perforatum, exhibits therapeutic potential against URSA, yet its underlying mechanism of action remains unknown. In this study, we employed an integrated multi‐omics approach coupled with a multi‐dimensional validation framework that spanned URSA patient decidual tissues, in vivo mouse models, and in vitro telomerase‐immortalized human endometrial stromal cell (T‐hESC) decidualization system, to systematically investigate hyperoside's mechanism in URSA, with a focus on R‐loop‐driven endometrial stromal cell senescence. We found that hyperoside dose‐dependently reduced embryo resorption and rescued decidualization deficiency by preventing stromal cell senescence. Mechanistically, hyperoside effectively alleviated aberrant intracellular R‐loop accumulation, thereby suppressing excessive activation of the cyclic GMP‐AMP synthase‐stimulator of interferon genes (cGAS‐STING) pathway which contributes to the initiation of the cellular senescence program. Further target identification and validation experiments confirmed that DExH‐box helicase 9 (DHX9) was the functional molecular target of hyperoside, with the Thr419 residue serving as the critical binding site. Functional validation revealed that DHX9 knockdown or introduction of the T419A point mutation markedly attenuated the anti‐senescence and pro‐decidualization effects of hyperoside. In vivo experiments further confirmed that uterine‐specific knockdown of DHX9 reduced hyperoside's protective effects against R‐loop accumulation, cGAS‐STING pathway activation, and embryo loss. Collectively, these findings demonstrate that hyperoside alleviates stromal cell senescence and decidualization deficiency in URSA through DHX9‐dependent resolution of R‐loops and subsequent suppression of cGAS‐STING‐associated senescence signaling. More broadly, this work identifies R‐loop‐mediated genomic stress as a previously underappreciated contributor to URSA‐associated decidual dysfunction and provides a mechanistic basis for the protective effects of hyperoside through DHX9‐dependent R‐loop homeostasis.

## Highlights

- Hyperoside alleviates endometrial stromal cell senescence, restores decidualization, and reduces embryo loss partly by mitigating pathological R‐loop accumulation.
- Integrated limited proteolysis‐mass spectrometry (Lip‐MS) and biophysical validation identify DExH‐box helicase 9 (DHX9) as a functional target of hyperoside, with Thr419 defined as a key residue required for effective hyperoside–DHX9 interaction.
- The study elucidates a novel pathogenic axis in unexplained recurrent spontaneous abortion (URSA) where R‐loop‐derived cytosolic DNA triggers cyclic GMP‐AMP synthase‐stimulator of interferon genes (cGAS‐STING) activation in stromal cells; hyperoside disrupts this cascade by resolving the upstream R‐loop trigger

## Graphical abstract

This study suggests that the food‐derived flavonoid hyperoside may act as a natural DExH‐box helicase 9 (DHX9) modulator, directly targeting Thr419 of DHX9 to help resolve pathological R‐loop accumulation. Consequently, it suppresses cyclic GMP‐AMP synthase‐stimulator of interferon genes (cGAS‐STING)‐driven endometrial stromal senescence and restores decidualization in unexplained recurrent spontaneous abortion (URSA). These findings indicate that R‐loop‐induced genomic stress represents a key mechanism in URSA, and position hyperoside as a mechanistically distinct candidate warranting further investigation.

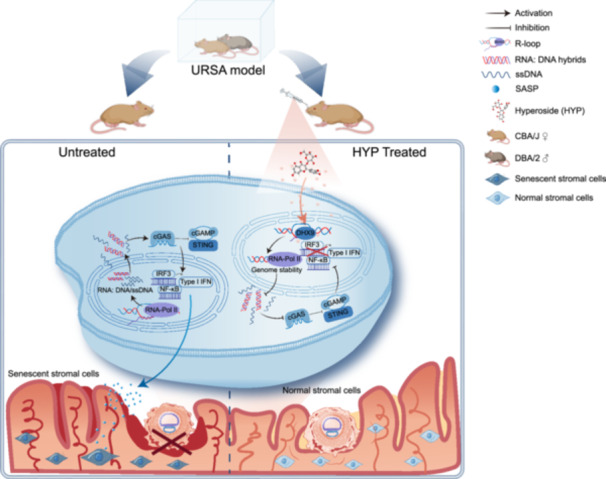

## INTRODUCTION

Recurrent spontaneous abortion (RSA) is defined as the occurrence of at least two consecutive pregnancy losses before the 24th week of gestation, affecting approximately 1% of reproductive‐aged couples and presents notable clinical challenges [1, 2]. Despite advances in identifying etiologies such as chromosomal defects, uterine malformations, and thrombophilias, nearly half of all RSA cases remain unexplained (unexplained recurrent spontaneous abortion, URSA) [1]. Emerging evidence highlights defective decidualization, a dynamic process in which endometrial stromal cells differentiate into decidual cells to support embryo implantation, as a critical contributor to URSA [3, 4, 5]. Compromised decidualization disrupts trophoblast invasion, immune tolerance, and placental development [3, 6, 7]; however, the molecular drivers of this dysfunction are incompletely understood. Cellular senescence, defined by non‐reversible cell cycle arrest triggered by intrinsic and extrinsic stressors, has emerged as a key mediator of endometrial dysfunction [8]. Extensive research has demonstrated that senescent endometrial stromal cells are unable to differentiate into decidual stromal cells and can secrete senescence‐associated secretory phenotype (SASP) factors that exacerbate inflammation, thus inhibiting decidualization and contributing to pregnancy loss [9]. Notably, senescent endometrial stromal cells were identified in the decidual tissue from patients with URSA [10], which may represent a pivotal mechanism underlying decidualization defects in this condition. Nevertheless, the precise upstream triggers initiating cellular senescence remain to be fully elucidated.

Mounting evidence suggests that persistent R‐loop accumulation is a key trigger of cellular senescence [11]. R‐loops represent triple‐stranded nucleic acid structures, consisting of RNA: DNA hybrids and unpaired single‐stranded DNA [12]. Under physiological conditions, transient R‐loop formation supports essential processes, including transcriptional regulation, chromatin remodeling, and immunoglobulin class switching [13, 14]. However, sustained R‐loop levels create transcription‐replication conflicts, inducing double‐strand DNA breaks, chromosomal instability, and epigenetic dysregulation, thus acting as key triggers of cellular senescence [12]. The latest findings have revealed that R‐loop‐derived cytosolic DNA may activate cyclic GMP‐AMP synthase (cGAS) to produce 2′3′ cyclic GMP‐AMP (cGAMP). cGAMP then binds stimulator of interferon genes (STING) [12, 15], triggering downstream signaling cascades. This cascade leads to activation of the interferon regulatory factor 3 (IRF3)‐mediated interferon (IFN) pathway and the nuclear factor‐kappa B (NF‐κB)‐induced SASP pathway [16, 17]. Crucially, SASP‐associated oxidative stress further amplifies R‐loop accumulation, creating a pathogenic feedback loop that exacerbates cellular senescence [18, 19]. Notably, the close association between R‐loops and cellular aging has been implicated in cancer [20], neurodegeneration [21], and autoimmune diseases [22]. Intriguingly, URSA patients exhibit pronounced R‐loop‐associated genomic stress signatures in decidual tissues, characterized by heightened DNA damage response (DDR) and SASP activation markers [9, 23, 24]‐phenotypes that are mechanistically consistent with R‐loop‐driven cGAS‐STING activation and cell senescence observed in other models. However, whether and how R‐loop accumulation initiates this self‐reinforcing senescence cascade in stromal cells of URSA remains undefined, representing a critical knowledge gap in understanding URSA pathogenesis.

Hyperoside (HYP), a flavonol glycoside naturally abundant in edible plants such as Hypericum perforatum (St. John's wort), Crataegus pinnatifida (hawthorn), and Vaccinium spp. (blueberries), has gained attention for its bioactive properties in inflammation and senescence‐related conditions [25]. As a dietary constituent, hyperoside is present in traditional herbal infusions and has been studied in preclinical models for its bioactivity in modulating inflammation and inhibiting oxidative stress [26, 27]. Furthermore, emerging evidence suggests that hyperoside may indirectly modulate genomic instability through its dual capacity to enhance DNA repair fidelity [28, 29]. Intriguingly, hyperoside also suppresses cytosolic DNA‐triggered cGAS‐STING activation under pathological degenerative conditions [30], a pathway mechanistically linked to R‐loop‐derived DNA leakage [12, 31]. Given its presence in common foods and its emerging role in regulating genomic stability, hyperoside represents a promising candidate compound to investigate in URSA, particularly through its potential to disrupt the R‐loop‐driven cGAS‐STING cascade in endometrial stromal senescence.

To address these unmet scientific questions, we adopted a translational research framework integrating URSA mouse models, telomerase‐immortalized human endometrial stromal cells (T‐hESCs), and clinical samples to elucidate the capacity of hyperoside to resolve R‐loop‐mediated genomic instability, suppress cGAS‐STING‐driven cellular senescence, and functionally restore endometrial stromal decidualization. Notably, mechanistic investigation uncovered that hyperoside directly binds to DExH‐box helicase 9 (DHX9) at Thr419—a key residue critical for helicase‐mediated R‐loop resolution—thereby facilitating nucleic acid hybrid dissociation. Importantly, this study presents the first evidence that pharmacological modulation of R‐loop homeostasis reverses endometrial dysfunction in URSA, unveiling a hitherto unrecognized molecular axis linking R‐loop homeostasis to pregnancy maintenance. By identifying hyperoside as a natural DHX9 modulator with anti‐senescence activity, our findings bridge the disciplines of phytochemical pharmacology and reproductive immunology, offering a mechanistically informed therapeutic strategy for decidualization deficiency in URSA.

## RESULTS

### Hyperoside alleviated decidualization dysfunction and decreased the embryo resorption rate in URSA mice

**FIGURE 1:**
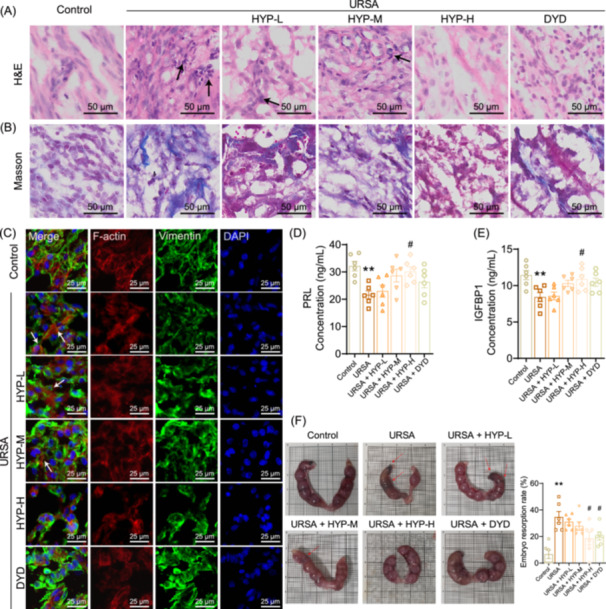
Hyperoside (HYP) alleviated decidualization dysfunction and decreased embryo resorption rate in unexplained recurrent spontaneous abortion (URSA) mice. (A) Representative hematoxylin and eosin (H&E) staining of decidua tissues from mice with control, URSA, low‐dose, medium‐dose, high‐dose hyperoside treatment, and dydrogesterone treatment. Black arrows indicate infiltrating inflammatory cells. Scale bar: 50 μm. (B) Representative Masson staining of decidua tissues from each group. The blue regions in Masson staining indicate collagen fiber deposition. Scale bar: 50 μm. (C) Rhodamine phalloidin staining of decidua tissues from each group showing the effect of hyperoside on vimentin‐positive cells' cytoskeletal integrity (White arrows indicate cytoskeletal disorganization). Scale bar: 25 μm. Enzyme‐linked immunosorbent assay (ELISA) was used to quantify the level of prolactin (PRL) (D) and insulin‐like growth factor binding protein 1 (IGFBP1) (E) in decidua tissues from each group (*n* = 6). (F) Embryo resorption in each group was recorded. The red arrow points to a representative resorbed fetus (*n* = 6). Data are presented as means ± SEM. Statistical significance is determined by one‐way ANOVA (Dunnett's post‐test). **p < 0.01 versus control group; ^#^ *p* < 0.05 versus URSA group.

Emerging clinical evidence highlights impaired decidualization as a key pathological mechanism in URSA, with abnormal morphology and secretory function strongly correlating with pregnancy loss [32, 33]. Translating these mechanistic insights into therapeutic discovery, we systematically evaluated hyperoside's effectiveness in restoring normal decidualization patterns in URSA model mice (Figure S1 presents the chemical structure, extracted ion chromatogram (EIC), and MS/MS spectrum of the hyperoside standard). Hematoxylin and eosin (H&E) staining revealed loss of regular stromal cell alignment, increased nuclear pleomorphism, and a higher density of infiltrating inflammatory cells in URSA decidua compared to controls, which were dose‐dependently ameliorated by hyperoside treatment (Figure 1A). Similarly, Masson staining demonstrated excessive collagen deposition in URSA decidua, indicative of fibrotic remodeling (Figure 1B). Both hyperoside and dydrogesterone interventions significantly attenuated extracellular matrix dysregulation, with hyperoside exhibiting a clear dose‐dependent efficacy (Figure 1B). Phalloidin‐based fluorescence imaging of F‐actin dynamics showed that hyperoside dose‐dependently ameliorated cytoskeletal disorganization, with a trend toward architectural normalization that was qualitatively similar to dydrogesterone treatment (Figure 1C). Furthermore, molecular analysis confirmed hyperoside's therapeutic effect on decidualization in URSA, with enzyme‐linked immunosorbent assay (ELISA) revealing significant recovery of prolactin (PRL) and insulin‐like growth factor binding protein 1 (IGFBP1) levels in treated groups (Figure 1D,E), aligning with the structural improvements. Additionally, hyperoside administration exhibited dose‐dependent therapeutic effects in lowering embryo resorption rates, with the highest protection observed in the high‐dose group (Figure 1F). These findings collectively demonstrate that hyperoside confers significant protection against URSA pathogenesis through dose‐responsive mechanisms.

### Hyperoside ameliorated stromal cell senescence in URSA

**FIGURE 2:**
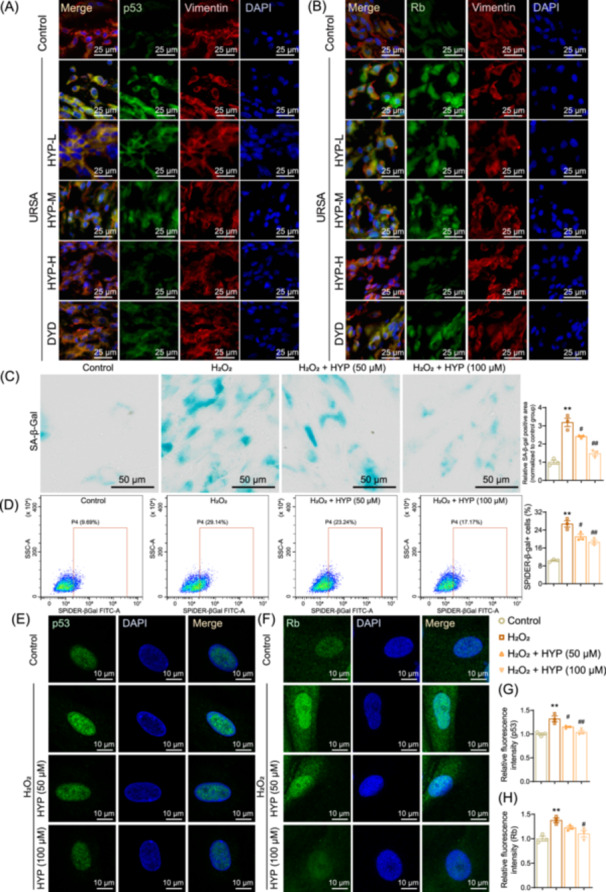
Hyperoside ameliorated stromal cell senescence in URSA mice and telomerase‐immortalized human endometrial stromal cells (T‐hESCs). (A, B) Immunofluorescence (IF) co‐staining was used to detect p53 (A), and Rb (B) in vimentin‐positive cells of decidua tissue from mice with control, URSA, low‐dose, medium‐dose, high‐dose hyperoside treatment, and dydrogesterone treatment. Scale bar: 25 μm. (C) Representative SA‐β‐gal images of T‐hESCs treated with H_2_O_2_ alone or H_2_O_2_ plus hyperoside (*n* = 3). Scale bar: 50 μm. (D) Flow cytometric analysis of SPiDER‐βGal^+^ cells treated with hyperoside (*n* = 3). The immunofluorescence intensity of p53 (E, G), and Rb (F, H) was detected by IF. Quantitative analysis and representative images of p53 (green), Rb (green), and nuclei (blue) are presented (*n* = 3). Scale bar: 10 μm. Data are presented as means ± SEM. Statistical significance is determined by one‐way ANOVA (Dunnett's post‐test). **p < 0.01 versus control group; ^#^ *p* < 0.05 versus H_2_O_2_ induction group; ^##^ *p* < 0.01 versus H_2_O_2_ induction group.

Building on established links between stromal cell senescence and decidualization failure [34], we first identified elevated expression of senescence biomarkers (γH2AX, p53, and Rb) in decidual stromal cells from URSA patients compared to healthy controls (Figure S2A–C), highlighting the clinical relevance of stromal senescence in this pathology. Recapitulating these human findings, the URSA murine model similarly showed increased levels of these markers (γH2AX, p53, and Rb) and higher fluorescence intensity in Vimentin‐positive cells compared to normal pregnancy controls (Figure 2A,B; Figure S2D–F). Concurrently, ELISA analysis revealed markedly elevated levels of SASP factors, including C‐X‐C motif chemokine ligand 1 (CXCL1) and interleukin‐6 (IL‐6), in URSA mice (Figure S2G,H). Furthermore, administering hyperoside effectively reduced this senescence phenotype in the URSA murine model, whereas dydrogesterone showed no significant effect, underscoring hyperoside's unique mechanism of action in alleviating stromal cell senescence in URSA (Figure 2A,B; Figure S2D–H).

To mechanistically dissect the action of hyperoside, we established a controlled in vitro system using T‐hESCs. Initial screening using CCK8 assays across a concentration series (0, 10, 20, 50, 100, and 150 μM) showed that 150 μM hyperoside significantly compromised cell viability, whereas lower concentrations exhibited no marked cytotoxicity (Figure S3A). Further assessment of SASP factors, including CXCL1 and IL‑6, revealed that hyperoside began to suppress their secretion at 50 μM, with a clear concentration‑dependent effect (Figure S3B,C). Based on these findings, we selected 50 and 100 μM for subsequent experiments. Notably, hyperoside dose‐dependently reduced cellular senescence, evidenced by decreased SA‐β‐Gal^+^ cells (Figure 2C) and confirmed through reduced SPiDER‐βGal^+^ populations in flow cytometry (Figures S3D; Figure 2D). Interrogation of molecular markers revealed hyperoside's capacity to mitigate genomic instability, as evidenced by a substantial reduction in γH2AX fluorescence intensity across treatment groups (Figure S3E). Quantitative fluorescence analysis revealed graded reductions in p53 and Rb signal intensity, correlating with restored equilibrium of senescence‐associated signaling (Figure 2E–H). Collectively, hyperoside suppressed stromal cell senescence responses in URSA in a concentration‐responsive manner, with maximal protective efficacy achieved at elevated dosage levels.

### Hyperoside attenuates stromal cell senescence through R‐loop homeostasis regulation

**FIGURE 3:**
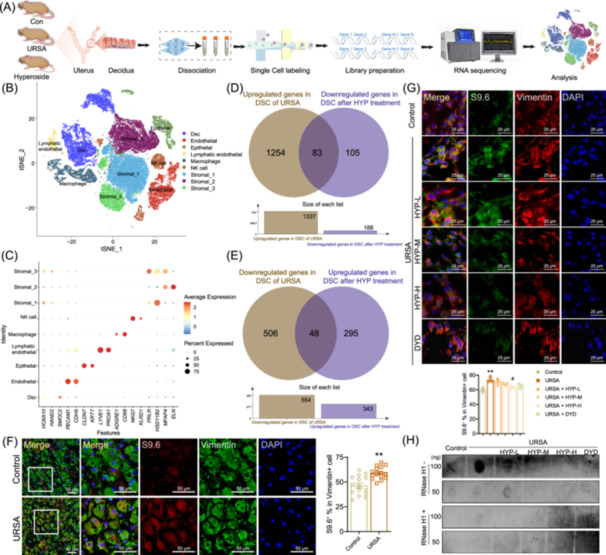
Hyperoside rescues stromal cell senescence in URSA by restoring R‐loop homeostasis. (A) Experimental workflow for single‐cell RNA sequencing. (B) tSNE visualization of cell clusters in control, URSA, and high‐dose hyperoside‐treated mice, colored by cell type. (C) Dot plot illustrating marker genes for individual cell subclusters. (D) Venn diagram illustrating the intersection of upregulated genes in decidual stromal cells (DSCs) of URSA and downregulated genes in DSCs following hyperoside treatment (83 overlapping genes). (E) Venn diagram illustrating the intersection of downregulated genes in DSCs of URSA and upregulated genes in DSCs following hyperoside treatment (48 overlapping genes). (F) IF co‐staining was used to detect S9.6 in vimentin‐positive cells from patients with normal pregnancy and URSA subjects (*n* = 15). Scale bar: 50 μm. (G) IF co‐staining was used to detect S9.6 in vimentin‐positive cells from mice with control, URSA, low‐dose, medium‐dose, high‐dose hyperoside treatment, and dydrogesterone treatment (*n* = 3). Scale bar: 25 μm. (H) Dot blot analysis to assess R‐loops in decidua from control, URSA, low‐dose, medium‐dose, high‐dose hyperoside treatment, and dydrogesterone treatment. Data are presented as means ± SEM. Statistical significance is determined by two‐tailed Student's *t* test or one‐way ANOVA (Dunnett's post‐test). **p < 0.01 versus control group; ^#^ *p* < 0.05 versus URSA group.

To address the unresolved upstream triggers of stromal cell senescence in URSA, we performed single‐cell RNA sequencing (scRNA‐seq) on decidual tissues from normal controls, URSA model mice, and hyperoside‐treated mice (Figure 3A). Based on lineage‐specific marker genes, 9 distinct cell types were identified, comprising decidual stromal cells (DSCs), endothelial cells, epithelial cells, stromal cells (type 1, type 2, and type 3), lymphatic endothelial cells, macrophage and NK cells, as the major cell types in mouse decidua (Figure 3B). Known and putative marker genes for all clusters are shown in Figure 3C. To further clarify the molecular landscape of DSC senescence, we focused on the profiles of differentially expressed genes (DEGs) in the DSC population. Among the identified DEGs, 1337 genes were upregulated in DSCs of URSA, and 188 genes were downregulated in DSCs after hyperoside treatment (Figure 3D). Intersecting the two gene sets revealed 83 common genes (Figure 3D). The enrichment analysis implied that hyperoside may alleviate decidual impairment and protect from URSA, potentially via regulation of RNA polymerase II transcription, regulation of mismatch repair, and in response to DNA damage (Figure S4A). In addition, 554 downregulated genes were identified in DSCs of URSA, while 343 upregulated genes were identified in DSCs after hyperoside treatment (Figure 3E). Venn diagrams showed 48 overlapping genes between downregulated genes in URSA group and upregulated genes in hyperoside treatment group (Figure 3E). Accordingly, the overlapped genes were enriched for facultative heterochromatin formation, apoptotic signaling pathway, and regulation to oxidative stress (Figure S4B). Overall, DEGs analysis and enrichment analysis revealed consistent pathways, including nucleic acid structure stabilization and the DDR, which are closely associated with R‐loop formation and resolution [35, 36]. These findings suggest that pathways governing R‐loop homeostasis are likely dysregulated in URSA and ameliorated by hyperoside treatment, reinforcing our hypothesis that hyperoside regulates stromal cell senescence through R‐loop homeostasis.

**FIGURE 4:**
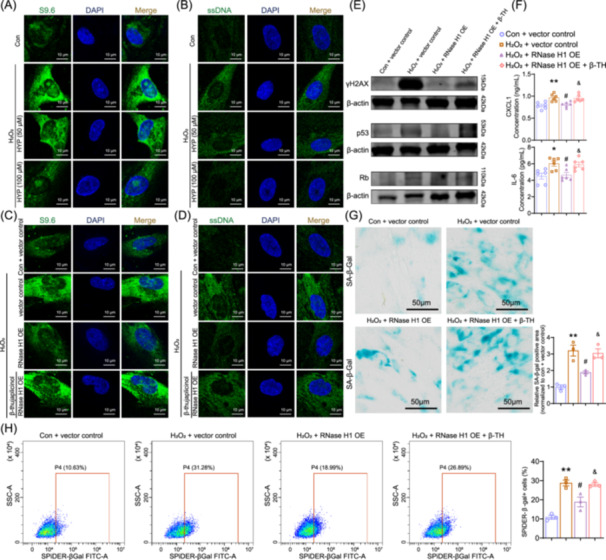
R‐loop accumulation contributes to cell senescence in T‐hESCs. (A, B) T‐hESCs were incubated with H_2_O_2_ and hyperoside (0, 50 µM, and 100 µM). After the intervention, the immunofluorescence intensity of S9.6 (A) and ssDNA (B) were detected by IF. Representative images of S9.6 (green), ssDNA (green), and nuclei (blue) are presented. Scale bar: 10 μm. IF staining was used to detect the immunofluorescence intensity of S9.6 (C), ssDNA (D) among groups. Representative images of S9.6 (green), ssDNA (green), and nuclei (blue) are presented. Scale bar: 10 μm. (E) Western blot was used to detect the expression of γH2AX, p53, and Rb among groups. (F) ELISA assay was used to detect the level of CXCL1 and IL‐6 from each group (*n* = 6). (G) Representative images of SA‐β‐Gal and quantitative analysis are presented (*n* = 3). Scale bar: 50 μm. (H) Flow cytometric analysis of SPiDER‐βGal^+^ cells from each group (*n* = 3). Data are presented as means ± SEM. Statistical significance is determined by one‐way ANOVA (Tukey post‐test). *p < 0.05 versus Con + vector control group; **p < 0.01 versus con + vector control group; ^#^ *p* < 0.05 versus H_2_O_2_ + vector control group; ^&^ *p* < 0.05 versus H_2_O_2_ + RNase H1 OE group.

Using S9.6 immunostaining, commonly used for recognizing RNA: DNA hybrids, we first identified significantly enhanced fluorescent co‐localization of S9.6 and Vimentin in decidual tissues from URSA patients (Figure 3F), suggesting pathological relevance in human pregnancy loss. This phenotype was recapitulated in URSA mice, where S9.6 signals were significantly elevated (Figure 3G). Hyperoside treatment attenuated these signals in a dose‐dependent manner, while dydrogesterone showed no effect (Figure 3G). Furthermore, dot blot analysis revealed that R‐loop levels were increased in URSA mice, while hyperoside induced a dose‐dependent decrease in these R‐loop levels (Figure 3H). In an H_2_O_2_‐induced senescence model of T‐hESCs, H_2_O_2_ treatment increased R‐loop accumulation and cytosolic single‐stranded DNA (ssDNA) levels compared to untreated controls (Figure 4A,B; Figure S4C). Hyperoside treatment significantly blunted these effects, with dose‐dependent reduction of S9.6 fluorescence intensity and cytosolic ssDNA levels (Figure 4A,B; Figure S4C).

To establish causality, we employed RNase H1 overexpression (RNase H1 OE) to degrade R‐loops in senescent T‐hESCs. Immunofluorescence analysis demonstrated that RNase H1 reduced H_2_O_2_‐induced R‐loop accumulation and ssDNA accumulation (Figure 4C,D; Figure S4D). Accordingly, RNase H1 overexpression alleviated DNA damage signaling (Figure S4E,F; Figure 4E) and senescence markers, including increased activation of the p53/Rb signaling pathway, SASP accumulation, SA‐β‐Gal^+^ cells, and SPiDER‐βGal^+^ cells (Figure 4E–H; Figure S4E,G,H). Conversely, in RNase H1‐overexpressing cells, co‐treatment with β‐thujaplicinol significantly restored R‐loop levels and exacerbated senescence markers (Figure 4C–H; Figure S4D–H). These data positioned R‐loop dysregulation as a critical node in hyperoside's anti‐senescence mechanism.

### cGAS‐STING is the key link for hyperoside to suppress senescence via R‐loop clearance

Having established hyperoside's therapeutic efficacy through R‐loop resolution, we next interrogated how this nucleic acid restructuring translates into senescence amelioration at the pathway level. To map the R‐loop‐regulated signaling network, we performed comparative transcriptomics between H_2_O_2_‐treated T‐hESCs with and without RNase H1 overexpression. The result identified 1974 DEGs between RNase H1‐overexpressing and vector control groups, including 886 upregulated and 1088 downregulated targets. Subsequent enrichment analysis of DEGs identified key GO‐BP terms, including regulation of type I interferon production and pattern recognition receptor signaling pathways (Figure S5A). Concurrently, KEGG pathway analysis highlighted enrichment in the Toll‐like receptor signaling cascade, NF‐κB pathway, and chemokine signaling network (Figure S5B). Collectively, these transcriptomic analyses revealed that R‐loop attenuation was associated with a broad downregulation of innate immune signaling pathways, including cytosolic DNA sensing and type I interferon responses. Given the central role of the cGAS‐STING axis in linking aberrant nucleic acid sensing to chronic inflammation and cellular senescence [37], we hypothesized that it might be a key mechanistic node underlying R‐loop‐driven stromal senescence in URSA. To test this, we next examined the activation status of the cGAS‐STING pathway in our clinical and experimental models. Clinical validation in human URSA decidual tissues demonstrated an increase in cGAS^+^ and STING^+^ fluorescence signals (Figure S5C,D). Similarly, immunofluorescence exhibited strong co‐localization of cGAS and STING with vimentin‐positive stromal cells, confirming pathway activation in the stromal compartment (Figure S6A,B). Importantly, hyperoside administration significantly diminished these signals (Figure S6A,B). In line with the above, ELISA showed that IFN‑β levels were markedly higher in URSA decidual tissues, whereas hyperoside treatment led to a pronounced decrease (Figure S6C).

To establish the causal link between R‐loop formation and cGAS‐STING pathway activation, we modulated R‐loop levels in H_2_O_2_‐treated T‐hESCs. Overexpression of RNase H1 significantly reduced both cGAS and STING levels (Figure S7A,B). Conversely, β‐thujaplicinol exacerbated cGAS/STING upregulation (Figure S7A,B). Together, these findings establish the R‐loop‐cGAS‐STING axis as the key mechanistic cascade by which hyperoside exerts its anti‐senescence efficacy.

### Hyperoside interacts with DHX9 at the key residue Thr419

**FIGURE 5:**
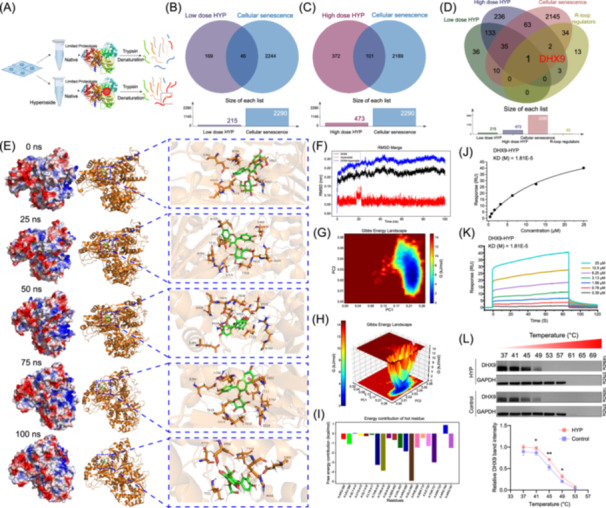
Hyperoside targets DExH‐box helicase 9 (DHX9) to resolve R‐loops and attenuate senescence in endometrial stromal cells via interaction at key residues. (A) Schematic diagram of the Limited proteolysis mass spectrometry (Lip‐MS) assay. (B) The Venn diagram showing the overlapping targets between senescence‐associated genes and the genes encoding proteins identified in low‐dose hyperoside‐treated cells (46 overlapping targets). (C) Venn diagram showing the overlapping targets between senescence‐associated genes and the genes encoding proteins identified in high‐dose hyperoside‐treated cells (101 overlapping targets). (D) Venn diagram of the corresponding genes of cell senescence‐HYP modulating protein candidates (46 from the low‐dose group and 101 from the high‐dose group) and R‐loop regulators. (E) Molecular docking validation of DHX9. Representative binding poses of hyperoside with DHX9 at 0 ns, 25 ns, 50 ns, 75 ns, and 100 ns. (F) Root‐mean‐square deviation (RMSD) merge plot of hyperoside in complex with DHX9 (blue: hyperoside‐DHX9 complex, black: DHX9, red: hyperoside). (G) Gibbs free energy landscape 2D of the hyperoside‐DHX9 complex. (H) Gibbs free energy landscape 3D of the hyperoside‐DHX9 complex. (I) The per‐residue energy contribution of hyperoside versus DHX9. The surface plasmon resonance (SPR) dynamic response resulting from the interaction of hyperoside‐DHX9 at a concentration of 0.39–25 μM: Steady state Affinity model fitting results, the *x*‐axis represents sample concentration, while the *y*‐axis represents binding signal (J), Surface‐bound hyperoside‐DHX9 profile (K). (L) CETSA analysis between hyperoside and DHX9 (*n* = 3). Data are presented as means ± SEM. Statistical significance is determined by two‐way ANOVA. *p < 0.05 versus Control group, **p < 0.01 versus Control group.

To investigate how hyperoside influences R‐loop dynamics and reduces endometrial stromal cell aging, we first used the limited proteolysis‐mass spectrometry (Lip‐MS) assay to identify its potential binding targets. Lip‐MS is an effective method for identifying potential targets of bioactive compounds. This method utilizes limited proteolysis to selectively break down proteins, generating peptide fragments for mass spectrometric analysis (Figure 5A). Using this approach, we treated URSA‐derived decidual stromal cells with two concentrations of hyperoside (50 and 100 μM). Lip‐MS profiling revealed 215 and 473 differentially abundant proteins in 50 μM hyperoside and 100 μM hyperoside groups, respectively. After mapping these proteins to their corresponding genes, intersection analysis with 2290 senescence‐associated genes yielded 46 and 101 overlapping genes (Figure 5B,C), which were further subjected to functional annotation. Strikingly, GO‐BP enrichment highlighted their collective involvement in DNA repair, recombinational repair, and KEGG pathways converged on apoptosis, cytosolic DNA sensing (Figure S8A–D). These findings strongly implicated hyperoside in modulating genomic stress pathways, particularly those linked to unresolved DNA lesions—a hallmark of cellular senescence.

To pinpoint the functional target of hyperoside in R‐loop regulation, we mapped the hyperoside‐modulated proteins to their corresponding genes (46 from the 50 μM group and 101 from the 100 μM group) and intersected them with cell senescence‐associated genes and R‐loop regulators (Table S1). This multi‐tiered screening strategy identified DHX9 (RNA helicase A) as the only overlapping target (Figure 5D), a helicase essential for resolving transcription‐replication conflicts by dismantling R‐loops and preventing DDR activation [38].

To determine the potential binding site of hyperoside within the DHX9 protein, we performed molecular docking simulations. Snapshots extracted at 0, 25, 50, 75, and 100 ns were analyzed. The results revealed that Thr419 consistently formed hydrogen bonds with hyperoside at all analyzed time points (Figure 5E). Then, molecular dynamics (MD) simulations of hyperoside ligands with DHX9 proteins were conducted to confirm the stability of their binding. Root‐mean‐square deviation (RMSD) analysis showed that the Hyperoside‐DHX9 (blue) exhibited higher fluctuations than DHX9 (black), suggesting that binding to hyperoside might induce conformational alterations (Figure 5F). The RMSD values remained within 1 nm throughout the simulation and achieved a relatively stable conformation after 20 ns. In addition, we accurately calculate the Gibbs free energy based on the RMSD and radius of gyration (Rg) values of the complex. As shown in Figure 5G,H, the free energy landscape (FEL) showed a nearly single and sharp minimum energy cluster, confirming the complex's stable thermodynamic state. The hydrogen bonds of DHX9‐hyperoside complex were in the range of 2–9, with discontinuous fluctuations at 10 or even 12 (Figure S8E). The high hydrogen bond density suggests that DHX9 forms stable interactions with hyperoside. The FEL, derived from RMSD and Rg, illustrates DHX9's stable conformation at the energy minimum cluster (Figure S8F,G). Additionally, to quantify the binding stability between hyperoside and the DHX9 protein receptor, we calculated the binding free energy and per‐residue energy contributions using the molecular mechanics‐generalized born surface area (MM‐GBSA) method. The per‐residue energy decomposition results are presented in Figure 5I. As shown in Table S2, the calculated binding free energy for the DHX9‐hyperoside complex was −50.86 ± 3.71 kcal/mol, indicative of a stable binding conformation. Combined with these results, Thr419 (T419) likely exhibits significant binding affinity towards hyperoside.

Furthermore, surface plasmon resonance (SPR) analysis quantitatively supported the interaction between hyperoside and DHX9, yielding a dissociation constant of 1.81E‐5 M (Figure 5J,K), suggesting a direct interaction between hyperoside and DHX9. To validate the critical role of T419, we assessed the binding of hyperoside to the DHX9‐T419A mutant (DHX9‐MUT). DHX9‐MUT was immobilized on CM5 chips at a density comparable to DHX9‐WT. Using the same analyte concentration series as for DHX9‐WT, no valid binding curve was obtainable for DHX9‐MUT (Figure S8H,I). A KD of >2.47 M was calculated by extending the fitted binding curve beyond the tested concentration range via instrument modeling, which indicates extremely weak binding between mutant DHX9 and hyperoside (Figure S8J,K). This drastic reduction in binding affinity relative to DHX9‐WT indicates that T419 is an essential binding residue for hyperoside recognition. Subsequently, cellular thermal shift assay (CETSA) was performed to evaluate the thermodynamic stability between hyperoside and DHX9, revealing a significant improvement in the stability of DHX9 in the hyperoside‐treated cells (Figure 5L). Collectively, these findings suggest a direct interaction between DHX9 and hyperoside, highlighting Thr419 as a critical residue for binding.

### Hyperoside suppresses R‐loop‐driven cGAS‐STING activation through functional modulation of DHX9

**FIGURE 6:**
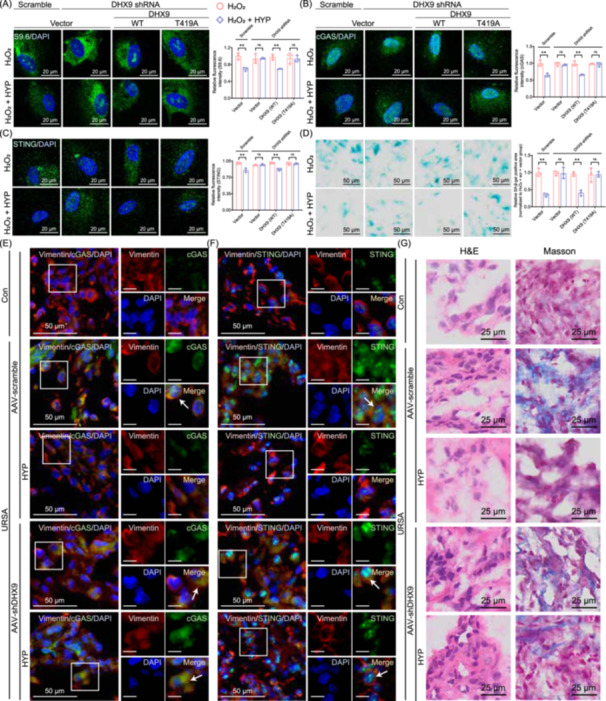
Hyperoside suppresses R‐loop‐driven cGAS‐STING activation via functional modulation of DHX9 both in vitro and in vivo. IF staining was used to detect the immunofluorescence intensity of S9.6 (A), cGAS (B), and STING (C) (*n* = 3). Scale bar: 20 μm. (D) Representative SA‐β‐gal images and analysis of each group (*n* = 3). Scale bar: 50 μm. IF co‐staining was used to detect cGAS (E) and STING (F) in the vimentin‐positive cells of decidua tissue from each group. White arrows indicate the co‐localization of cGAS, and STING with vimentin. Scale bar: 50 μm (left), 10 μm (right). (G) Representative H&E and Masson staining of decidua tissues from each group. Scale bar: 25 μm. Data are presented as means ± SEM. Statistical significance is determined by two‐way ANOVA. **p < 0.01; ns, Non‐significant.

To establish DHX9 as the molecular target through which hyperoside modulates R‐loop‐dependent senescence pathways, we performed systematic functional validation. Initial experiments confirmed that hyperoside treatment (100 μM) did not alter DHX9 protein abundance (Figure S9A), indicating that hyperoside modulates DHX9 function independently of its expression level. To definitively establish that hyperoside's therapeutic effects rely on DHX9 activity, we performed genetic reconstitution experiments. Quantitative analysis revealed that hyperoside significantly attenuated H_2_O_2_‐induced senescence markers in T‐hESCs. Immunofluorescence detection using the S9.6 antibody showed a significant reduction in R‐loop accumulation. Besides, the inhibitory effects of hyperoside extended to the cGAS/STING signaling axis, γH2AX levels, p53/Rb cell cycle regulatory pathway, and SA‐β‐Gal enzymatic activity assessed by senescence‐associated β‐galactosidase kit staining (Figure 6A–D; Figure S9B–D). Critically, DHX9 knockdown (shDHX9) largely compromised these protective effects, as hyperoside failed to reduce senescence parameter in DHX9‐knockdown cells (Figure 6A–D; Figure S9B–D). Functional rescue experiments established mechanistic specificity. Reconstitution with wild‐type DHX9 (DHX9‐WT) restored hyperoside's capacity to suppress all measured markers: R‐loops, cGAS/STING signaling, γH2AX, p53/Rb, and SA‐β‐Gal positivity (Figure 6A–D; Figure S9B–D). In striking contrast, cells expressing DHX9‐T419A mutant remained refractory to hyperoside treatment, exhibiting no significant reduction in senescence markers despite exposure to the drug (Figure 6A–D, Figure S9B–D). This unequivocally demonstrates that both DHX9 expression and its helicase activity are essential for hyperoside's anti‐senescent function.

To establish that DHX9 is essential for hyperoside's action in vivo, we first knocked down DHX9 in the endometrium of URSA‐model mice using a uterine‐injected, AAV‐shDHX9. Immunofluorescence staining of uterine sections from AAV‐shDHX9‐injected mice showed that DHX9 expression was markedly reduced in vimentin‐positive stromal cells, confirming knockdown in stromal cells (Figure S10A,B). We then compared the effects of hyperoside treatment in mice receiving either a control shRNA (AAV‐scramble) or the DHX9‐targeting shRNA (AAV‐shDHX9). This design allowed us to test whether DHX9 knockdown substantially reduced the therapeutic benefits of hyperoside. Strikingly, S9.6^+^ R‐loop accumulation persisted in the decidual stromal compartment of hyperoside‐treated DHX9‐knockdown mice (Figure S10C,D), indicating impaired resolution of transcription‐replication conflicts. Furthermore, hyperoside failed to suppress cGAS‐STING pathway activation (Figures S10D; Figure 6E,F), reduce γH2AX, p53, and RB levels (Figure S10E–J), or normalize CXCL1/IL‐6 secretion (Figure S10K) in DHX9‐knockdown mice. Histologically, in the DHX9 knockdown group, F‐actin cytoskeletal organization was disrupted despite hyperoside treatment (Figure S10L). Furthermore, H&E staining revealed disorganized decidual architecture, and Masson staining showed excessive collagen deposition (Figure 6G). This was accompanied by decreased levels of PRL and IGFBP1 (Figure S10M) and elevated embryo resorption rates (Figure S10N). Collectively, these findings indicate that hyperoside lost its therapeutic efficacy in the absence of functional DHX9.

**FIGURE 7:**
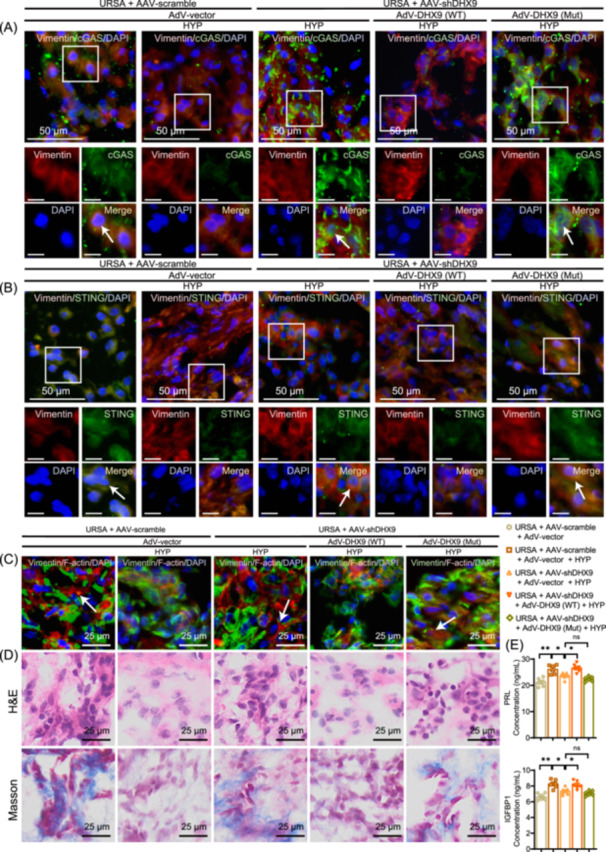
Hyperoside exerts its therapeutic effects in mice by targeting the T421 residue of DHX9. IF co‐staining was used to detect cGAS (A) and STING (B) in the vimentin‐positive cells of decidua tissue from each group. White arrows indicate the co‐localization of cGAS, and STING with vimentin. Scale bar: 50 μm (up), 10 μm (down). (C) Rhodamine phalloidin staining of the vimentin‐positive cells in decidua tissue from each group. White arrows indicate cytoskeletal disorganization. Scale bar: 25 μm. (D) Representative H&E and Masson staining of decidua tissues from each group. Scale bar: 25 μm. (E) ELISA assay was used to detect the level of PRL and IGFBP1 from each group (*n* = 6). Data are presented as means ± SEM. Statistical significance is determined by one‐way ANOVA (Tukey post‐test). *p < 0.05; **p < 0.01; ns, Non‐significant.

To further confirm that hyperoside's effects depend on DHX9, we conducted rescue experiments. When AAV‐shDHX9 mice were treated with hyperoside alongside an adenovirus encoding wild‐type DHX9, we observed striking improvements: wild‐type DHX9 reintroduction reversed R‐loop accumulation (Figure S11A,B), restored hyperoside‐mediated suppression of cGAS‐STING activation (Figure 7A,B; Figure S11B), reduced γH2AX/p53/RB levels (Figure S11C–H), and normalized CXCL1/IL‐6 secretion (Figure S11I). Histologically, wild‐type DHX9 rescue restored F‐actin cytoskeletal organization (Figure 7C), improved decidual morphology (H&E) and reduced fibrosis (Masson staining) (Figure 7D), and elevated PRL/IGFBP1 levels (Figure 7E). In contrast, mice expressing the T421A mutant (Mut) exhibited persistent R‐loop accumulation, cellular senescence, and tissue damage despite hyperoside treatment, phenocopying DHX9‐knockdown mice. These findings collectively demonstrate that hyperoside requires functional DHX9 (with the critical residue corresponding to T421 in mice) to attenuate R‐loop/cGAS‐STING‐mediated senescence in URSA stromal cells, thereby supporting decidualization and pregnancy maintenance.

## DISCUSSION

URSA remains a therapeutic enigma, with current strategies such as progesterone supplementation primarily addressing hormonal imbalances rather than underlying cellular dysfunction [39, 40]. Emerging evidence suggests that impaired decidualization arises from stromal cell senescence triggered by unresolved genomic stress [8, 10], highlighting the need for interventions targeting these root causes. Here, we identified that hyperoside, a naturally occurring flavonoid in edible plants, as a candidate to mitigate URSA by resolving R‐loop‐driven genomic instability and suppressing cGAS‐STING‐mediated senescence, thereby restoring decidual homeostasis. These findings bridge dietary bioactivity and reproductive health, positioning hyperoside as a preclinical natural product candidate with potential relevance to decidual dysfunction.

Building on the paradigm shift toward targeting senescence‐driven decidual dysfunction, our study identifies that hyperoside exerts its therapeutic effect in URSA by resolving a pivotal driver of stromal senescence: R‐loop dysregulation. R‐loops, three‐stranded RNA: DNA hybrids traditionally viewed as transcriptional byproducts, have emerged as critical contributors to genomic instability and cellular senescence‐associated disease [12, 41]. For instance, Krishnan et al. reported that RNF8 deficiency in human BRCA1‐mutant breast cancer cells leads to the formation of R‐loop and an unstable replication fork, which exacerbates DNA damage, senescence, and synthetic lethality [42]. Studies have demonstrated that MCM8 deficiency, a key gene in reproductive aging, disrupts its interaction with helicases DDX5/DHX9, which may lead to unresolved R‐loops and DNA damage, ultimately resulting in reproductive reserve depletion and age‐related fertility decline [43]. Although R‐loop dynamics are implicated in various pathologies, their role and the potential of targeting their resolution machinery in URSA remained unexplored. Our data provide the first evidence that hyperoside alleviates URSA‐associated decidualization failure by directly counteracting aberrant R‐loop accumulation in endometrial stromal cells—a phenotype mechanistically consistent with R‐loop‐driven senescence observed in cancer [20] and other reproductive failures [43]. Notably, in our H_2_O_2_‐induced stromal senescence model, hyperoside‐mediated R‐loop resolution significantly attenuated DNA damage signaling (γ‐H2AX), cell cycle blockade (p53 and Rb), and SASP secretion. Therefore, hyperoside's ability to suppress R‐loops represents a novel and effective strategy to mitigate stromal cell senescence and restore decidualization in URSA, positioning it as a key mechanism underlying its therapeutic efficacy.

DHX9, also known as RNA helicase A (RHA), is an essential component of the RNA polymerase II (Pol II) holoenzyme, which facilitates co‐transcriptional pre‐mRNA processing through its helicase activity [44, 45]. Specializing in resolving aberrant nucleic acid structures, like DNA: RNA hybrids and G‐quadruplexes (G4), DHX9 is crucial for maintaining R‐loop homeostasis [46]. Disruption of this equilibrium is mechanistically linked to genomic instability and aging‐related pathologies, as evidenced by studies showing that DHX9 depletion induces premature senescence in mouse fibroblasts, which leads to aberrant R‐loop metabolism, further DNA replication stress, and subsequent senescence [38]. Notably, our findings reveal that hyperoside binds to Thr419 of human DHX9, a residue evolutionarily conserved as Thr421 in murine DHX9, thereby eliciting a potent anti‐senescence effect. This interaction modulates DHX9 bioactivity, significantly inhibiting R‐loop formation and associated DNA damage effects that align with the observed anti‐senescence phenotype in URSA stromal cells. This mechanism diverges fundamentally from conventional anti‐senescence strategies, which focus on ROS scavenging or SASP inhibition [47, 48]. Instead, hyperoside addresses senescence at its upstream trigger by resolving R‐loop‐derived genomic stress, representing a prophylactic rather than a palliative therapeutic approach.

Additionally, central to hyperoside's therapeutic cascade is its capacity to disrupt the R‐loop‐cGAS‐STING signaling axis by interacting with DHX9. Our mechanistic dissection reveals that cGAS‐STING activation serves as the indispensable signaling node that converts accumulated R‐loops into inflammatory senescence, with hyperoside's DHX9 Thr419‐binding effectively decoupling this pathological relay. Cytosolic DNA fragments derived from unresolved R‐loops exhibit high affinity for the cGAS DNA‐binding domain, triggering phase separation that enhances STING oligomerization [49, 50]. This structural rearrangement enables TANK‐Binding Kinase 1 (TBK1)‐mediated phosphorylation at Ser366, thereby activating both the IRF3‐driven interferon response and NF‐κB‐mediated SASP amplification [51, 52], which may sustain a proinflammatory decidual microenvironment that perpetuates placental dysfunction and leads to miscarriage [53, 54]. Our discovery that R‐loop‐dependent cGAS‐STING activation drives URSA pathogenesis aligns with recent studies, which have shown that R‐loop accumulation triggers cGAS activation through the release of cytosolic DNA [55]. Similarly, hyperoside's ability to suppress cGAS signaling has been documented in neuroinflammation models [30]. Furthermore, our work extends these observations by identifying DHX9‐mediated R‐loop resolution as the upstream regulatory mechanism, a novel contribution to the field. Notably, this study first demonstrated that cGAS‐STING was significantly upregulated in URSA decidua tissue, which was regulated in a DHX9‐dependent manner via R‐loop modulation, a process that could be therapeutically targeted by hyperoside. This DHX9‐R‐loop‐cGAS‐STING axis represents a previously unrecognized pathway in reproductive senescence, distinct from but complementary to known cGAS‐STING activation mechanisms in aging [56] and autoimmune diseases [57]. Collectively, by disrupting the R‐loop‐cGAS‐STING‐SASP feedforward loop, hyperoside not only rescues decidual function but also redefines URSA as a disorder of transcriptional‐replication conflict resolution—a paradigm shift with broad implications for senescence‐targeted therapies in reproductive medicine.

Clinically, hyperoside's dose‐dependent efficacy in restoring decidual markers and reducing embryo resorption rates, comparable to dydrogesterone but with superior senescence‐targeting activity, supports its potential as a preclinical candidate in URSA management. These results not only position it as a promising alternative to hormonal therapies but also highlight its translational promise due to its dual capacity to resolve genomic instability and attenuate inflammation, a combination unaddressed by existing URSA therapies. This aligns with recent evidence that enhancing endometrial receptivity by suppressing cell cycle arrest is a crucial pathway for improving decidual aging [34]. However, to accelerate clinical translation, future studies should prioritize pharmacokinetic profiling of hyperoside in pregnancy‐compatible models and explore synergies with progesterone to enhance decidual resilience. Additionally, the interplay between hyperoside and progesterone receptor (PR) signaling warrants exploration, as dysregulated PR signaling is a known contributor to recurrent miscarriage, particularly in cases associated with decidualization deficiency [58, 59]. Besides, exploring DHX9 polymorphisms in URSA cohorts is necessary, which may help personalize dietary advice, as helicase activity variations could influence responsiveness to hyperoside‐rich foods. Such combinatorial approaches may yield additive benefits, particularly in URSA cases with concurrent hormonal and cellular pathologies.

Several limitations should be acknowledged. First, although we identified DHX9 Thr419 as the critical binding residue for hyperoside, the precise molecular mechanism by which hyperoside binding enhances DHX9 helicase activity, such as inducing conformational changes or relieving autoinhibition, remains to be elucidated. Second, while we identified DHX9 as a key target, hyperoside may have additional targets that contribute to its effects. Third, the clinical safety and efficacy of hyperoside must be validated in expanded cohorts of pregnant women to enable clinical translation. Despite these limitations, our multi‐omics and functional data provide a robust mechanistic foundation for hyperoside's therapeutic effects in URSA.

## CONCLUSION

In summary, this research unveils hyperoside as a natural compound targeting R‐loop‐driven senescence to ameliorate URSA. By elucidating the R‐loop‐cGAS‐STING axis as a mechanistic linchpin, our findings advance the understanding of decidualization pathology and provide a roadmap for developing phytochemicals against pregnancy‐related senescence disorders. We demonstrate that hyperoside repositions URSA therapeutics from hormonal support to precision targeting of senescence‐driving R‐loops, resolving transcription‐replication conflicts—an underexplored axis in reproductive medicine. These results not only reinforce the therapeutic value of traditional medicinal plants but also highlight the untapped potential of RNA: DNA hybrid biology in addressing multifactorial reproductive pathologies. By pioneering a phytochemical strategy that bridges genomic instability resolution and inflammation suppression, this study highlights the transformative role of plant‐derived compounds in modern medicine and offers a paradigm for combating complex gestational disorders.

## METHODS

### Reagents and antibodies

The following reagents and materials were used in this study: Hyperoside (Yuanye Bio‐Technology, Cat. No.: B20631), dydrogesterone (MCE, Cat. No.: HY‐B0257A), β‐thujaplicinol (MCE, Cat. No.: HY‐W060316), H_2_O_2_ (Sigma‐Aldrich, Cat. No.: 88597), 8‐bromoadenosine 3′, 5′‐cyclic monophosphate (8‐Br‐cAMP) (Sigma, Cat. No.: B5386), Medroxyprogesterone acetate (MPA) (APExBIO, Cat. No.: B1510), DMEM/F12 (Thermo Fisher, Cat. No.: 12634010), Fetal bovine serum (FBS) (Gibco, Cat. No.: 10099141), Penicillin (Beyotime, Cat. No.: ST488‐1), Streptomycin (Beyotime, Cat. No.: ST488‐2); rAAV‐U6‐shRNA (DHX9)‐CMV‐3Xflag‐SV40 polyA (BrainVTA Co., Ltd., Wuhan, China), rAAV‐U6‐shRNA (scramble)‐CMV‐3Xflag‐SV40 polyA (BrainVTA Co., Ltd., Wuhan, China), LV‐U6‐shRNA (DHX9)‐CMV‐3XFlag‐T2A‐Puro‐WPRE (BrainVTA Co., Ltd., Wuhan, China), LV‐U6‐shRNA (Scramble)‐CMV‐3XFlag‐T2A‐Puro‐WPRE (BrainVTA Co., Ltd., Wuhan, China); DHX9 (TargetMol, Cat. No.: TMPH‐00974), DHX9 mutant (T419A) (General biol Co., Ltd., Chuzhou, China); Senescence β‐Galactosidase Staining Kit (Beyotime, Cat. No.: C0602), SPIDER‐βGal Kit (Dojindo, Cat. No.: SG03), CoraLite®594‐Phalloidin (Proteintech, Cat. No.: PF00003); Mouse IL‐6 ELISA kit (BYabscience, Cat. No.: BY‐EM220188), Mouse CXCL1 ELISA kit (BYabscience, Cat. No.: BY‐EM220048), Mouse PRL ELISA kit (BYabscience, Cat. No.: BY‐EM220246), Mouse IGFBP1 ELISA kit (BYabscience, Cat. No.: BY‐EM228061), Mouse IFN‐β ELISA kit (BYabscience, Cat. No.: BY‐EM220131), Human IL‐6 ELISA kit (BYabscience, Cat. No.: BY‐EH110377), Human CXCL1 ELISA kit (BYabscience, Cat. No.: BY‐EH112946). The following primary and secondary antibodies were utilized in this study: Rabbit anti‐Phospho‐Histone H_2_AX (Ser139) (γH2AX) (Cell Signaling Technology, Cat. No.: 2577), rabbit anti‐p53 (Abcam, Cat. No.: ab131442), rabbit anti‐Rb (Abcam, Cat. No.: ab181616), rabbit anti‐DHX9 (Cell Signaling Technology, Cat. No.: 70998), rabbit anti‐β‐actin (Cell Signaling Technology, Cat. No.: 4970), rabbit anti‐Vimentin (Abcam, Cat. No.: ab92547), mouse anti‐Vimentin (Abcam, Cat. No.: ab8978), mouse anti‐DNA‐RNA Hybrid Antibody (S9.6) (AntibodySystem, Cat. No.: RGK60001), mouse anti‐ssDNA (Sigma, Cat. No.: ZMS1042), rabbit anti‐cGAS (Abcam, Cat. Nos.: ab302617 and ab252416), rabbit anti‐STING (Cell Signaling Technology, Cat. No.: 13647); Goat anti‐rabbit IgG (H&L) (LI‐COR, Cat. No.: 926‐32211), goat anti‐rabbit IgG H&L (Alexa Fluor® 488) (Abcam, Cat. No.: ab150077), goat anti‐rabbit IgG H&L (Alexa Fluor® 647) (Abcam, Cat. No.: ab150083), goat anti‐mouse IgG H&L (Alexa Fluor® 488) (Abcam, Cat. No.: ab150113).

### Patients and sample collection

The project adhered to the principles outlined in the Declaration of Helsinki. Ethics approval for this study involving human tissues was granted by the Ethics Committee of Hangzhou Hospital of TCM, affiliated with ZCMU (Hangzhou, China) (No. 2024KLL119). Written informed consent was obtained from all participants. Decidual tissues were collected from two cohorts: the URSA group (patients with ≥2 consecutive losses before 12 weeks, after exclusion of known etiologies including chromosomal abnormalities, uterine malformations, antiphospholipid syndrome, thrombophilias, and endocrine disorders) and the Control group (healthy women with at least one prior live birth undergoing elective surgical pregnancy termination for social reasons) (*n* = 15 per group). Following vacuum aspiration, all specimens were immediately rinsed with sterile ice‐cold phosphate‐buffered saline (PBS) and subsequently processed or preserved according to specific experimental protocols. Both groups were matched for maternal age, the gestation period, and BMI at sampling. Table S3 summarized the baseline cohort characteristics. In addition, primary decidual stromal cells were isolated from the decidual tissues of the URSA group by collagenase digestion and Percoll gradient purification as previously described [60], and cultured in DMEM with 10% FBS.

### Animal model

A group of 6‐week‐old female CBA/J, male BALB/c, and male DBA/2 mice was obtained from Huafukang Biotechnology. All specific pathogen‐free grade mice were acclimated for 1 week in a standard environment characterized by a constant temperature of 21°C–23°C and humidity of 50%–60%, with free access to adequate food and water. The animal experiments were approved by the Institutional Animal Care and Use Committee at Zhejiang Chinese Medical University (No. IACUC‐202505‐13). Female CBA/J mice were crossed with male BALB/c mice and male DBA/2 mice at a 2:1 ratio to create either a normal pregnancy model or a model prone to spontaneous abortion. The day of vaginal plug detection was defined as gestational day (GD) 0.5. Although the high pregnancy failure rate in the CBA/J × DBA/2 combination is often attributed to immune‐mediated fetal rejection, previous studies have also demonstrated decidualization defects as a contributing factor to pregnancy loss in this model [60, 61]. These findings further support the relevance of this model for studying the underlying mechanisms of decidualization deficiency in URSA.

### Experimental design and treatment strategies

Study on the therapeutic efficacy of hyperoside in URSA mice: Partial model mice were randomly selected to receive intragastric administration of low‐dose hyperoside (9 mg/kg), medium‐dose hyperoside (18 mg/kg), high‐dose hyperoside (36 mg/kg), and dydrogesterone (3.03 mg/kg) starting at GD 0.5. All mice were euthanized in two batches, on GD 8.5 and GD 14.5, respectively. All mice, regardless of treatment group, were euthanized following the same schedule and method. Their uteri, embryos, placenta, and decidua were dissected and prepared for subsequent analyses. All tissues were stored at −80°C or fixed in 4% paraformaldehyde. Additionally, the embryo resorption rate was computed using the formula: (number of resorbed implantation sites/total implantation sites) × 100%.

The molecular weight and purity level of the hyperoside were 464.38 g/mol and ≥98%, and were validated using liquid chromatography‐mass spectrometry (LC‐MS) (Figure S1).

Functional validation of DHX9 in hyperoside‐mediated suppression of the R‐loop‐driven cGAS‐STING in mice: Animals were randomly assigned to the following five groups (*n* = 12 per group): (1) Control + AAV‐scramble, (2) URSA + AAV‐scramble, (3) URSA + AAV‐scramble + HYP, (4) URSA + AAV‐shDHX9, (5) URSA + AAV‐shDHX9 + HYP.

Exploring the target effect of hyperoside on the T421 residue of DHX9 in the URSA murine model: Animals were randomly assigned to the following five groups (*n* = 12 per group): (1) URSA + AAV‐scramble + AdV‐vector, (2) URSA + AAV‐scramble + AdV‐vector + HYP, (3) URSA + AAV‐shDHX9 + AdV‐vector + HYP, (4) URSA + AAV‐shDHX9 + AdV‐DHX9 (WT) + HYP, (5) URSA + AAV‐shDHX9 + AdV‐DHX9 (Mut) + HYP.

### Determination of HYP by LC‐MS

Hyperoside (1 mg) was dissolved in 1 mL methanol as a 1 mg/mL stock solution, which was diluted to 20,000 ng/mL. Analysis was performed on an Agilent 1290 UHPLC coupled with an AB Sciex QTOF 5600 system. Chromatographic separation was achieved on a Waters Acquity HSS T3 column (2.1 × 100 mm, 1.8 µm) at 40°C. The mobile phase consisted of 0.1% formic acid water (A) and acetonitrile (B) with a gradient of 5%–20% B (0–5 min), 20%–55% B (5–10 min), 55%–70% B (10–11.5 min), 70%–95% B (11.5–12 min), 95%–5% B (12–12.5 min), at 0.3 mL/min (injection: 5 µL). MS detection was performed in ESI+ mode with TOF MS‐IDA‐Product Ion scan. Source parameters: ion spray voltage +5500 V, temperature 450°C, CUR 30 psi, GS1 55 psi, GS2 55 psi, and DP 60 V.

### DHX9 gene interference sequence screening

In this study, we employed a series of plasmids to achieve targeted gene silencing in 293T cells. Our primary goal was to identify the most effective plasmid configuration for maximizing DHX9 suppression. To address this, we transfected 293T cells with these plasmids and conducted each experimental condition in triplicate to ensure the reliability and reproducibility of our results. Following transfection, we extracted total RNA from the cells and performed quantitative real‐time PCR to systematically evaluate the inhibitory effects of each plasmid on DHX9 expression. Among the plasmids tested, the DHX9 shRNA1 (LV) construct achieved the most significant reduction in DHX9 expression, outperforming other interfering constructs in terms of knockdown efficiency (Figure S12A). The plasmid sequences are provided in detail within Table S4. Similarly, we applied the same method for the DHX9 shRNA (AAV) plasmids in B16 cells. The DHX9 shRNA2 (AAV) construct demonstrated the most significant reduction in DHX9 expression, outperforming other interfering constructs in terms of knockdown efficiency (Figure S12B). The plasmid sequences are provided in detail within Table S5. These two optimal plasmids were subsequently used for LV and AAV packaging, respectively.

### Adeno‐associated virus (AAV)‐mediated DHX9 knockdown combined with adenovirus (AdV) rescue experiments

To achieve optimal gene expression timing in our URSA model, we conducted local uterine injections of the rAAV‐U6‐shRNA2 (DHX9)‐CMV‐3Xflag‐SV40 polyA vector 3 weeks prior to model preparation. Isoflurane‑anesthetized mice were disinfected with 75% alcohol on the lower abdomen. A longitudinal incision allowed exteriorization of the uterus, which was then injected with AAV at multiple points based on the grouping. After injection, the uterus was carefully repositioned, and the abdominal wound was sutured to close the incision. Knockdown efficiency in vimentin‐positive stromal cells was validated by immunofluorescence (Figure S10A,B). This approach ensured that the AAV vector had sufficient time to achieve optimal gene expression before the URSA model was established. On Day 20 after AAV injection, AdV infection was initiated to perform rescue experiments, with mice divided into groups receiving either wild‐type (WT) AdV (ADV‐CMV‐mDHX9 WT‐P2A‐3Xflag‐polyA) or the T421A mutant AdV (ADV‐CMV‐mDHX9 T421A‐P2A‐3Xflag‐polyA) (homologous to human DHX9 T419) following standard viral transduction protocols. This design aimed to verify whether the phenotypic changes induced by DHX9 knockdown could be reversed by functional compensation of AdV‐mediated gene expression: specifically, the wild‐type AdV was used to restore normal DHX9‐related functions, while the T421A mutant AdV served as a control to assess the specificity of the rescue effect, helping to distinguish between specific functional recovery and non‐specific viral effects.

### Cell culture and treatment

Human telomerase reverse transcriptase‐immortalized human endometrial stromal cells (T‐hESCs, ATCC, Cat. No.: CRL‐4003) were cultured in DMEM/F12 medium, which was supplemented with 10% FBS and 1% penicillin‐streptomycin. These cells were grown under controlled conditions at 37°C with 5% CO_2_. Decidualization was induced by exposing the T‐hESCs to 1 μM MPA and 0.5 mM 8‐Br‐cAMP for 6 days. On the fourth day of decidualization, the cells were prepared for subsequent experiments. To mimic the oxidative stress‐driven senescence observed in the RSA decidualization defect, T‐hESCs were exposed to 200 µM H_2_O_2._ To investigate the causal relationship between R‐loops and cellular senescence, T‐hESCs were transfected with an RNase H1 overexpression plasmid. Conversely, T‐hESCs were treated with β‐thujaplicinol to counteract RNase H1 overexpression. To knock down the DHX9 expression, the cells were transduced with lentivirus expressing DHX9 shRNA or scramble shRNA. To perform rescue experiments, shDHX9 T‐hESCs were treated with DHX9 (WT) or DHX9 mutant (T419A). T‐hESCs were seeded in 96‐well plates and treated with hyperoside at the indicated concentrations. Then, 10 μL of CCK‐8 solution was added to each well and incubated for 2 h at 37°C. Absorbance was measured at 450 nm using a microplate reader.

### Western blot analysis

The expressions of β‐actin, γH2AX, p53, Rb and DHX9 in decidual tissues or cells were detected by Western blot. Decidua tissue or cell lysates were processed, then electrophoresis and membrane transfer were performed (280 mA, 90 min). The membranes were then incubated with γH2AX, p53, Rb, cGAS, STING, DHX9, and β‐actin antibodies, followed by the secondary antibodies. Following the scanning of the membranes using the Odyssey fluorescence imaging system.

### Immunofluorescence and confocal microscopy

Immunofluorescence staining was performed on decidual tissues or cells cultured on glass coverslips. First, fixed, permeated, and blocked tissues or cells. Subsequently, the slides were incubated with primary antibodies at 4°C overnight (γH2AX, p53, Rb, Vimentin, S9.6, ssDNA, cGAS, STING, 1:200 dilution). The next day, the slides were treated with secondary antibodies for 1 h at room temperature. Finally, the slides were mounted and examined using a fluorescence microscope (VS120‐S6‐W, Olympus) or a laser confocal microscope (LSM880, Zeiss).

### Morphological evaluation of decidualization

In this study, the distribution of F‐actin was investigated to evaluate decidualization using fluorescently labeled phalloidin, which specifically binds to F‐actin. First, fixed, permeated, and blocked tissues or cells, and then added fluorescence‐labeled phalloidin working solution, and incubated them for 20 min. Then, a fluorescence microscope (VS120‐S6‐W, Olympus) or a laser confocal microscope (LSM880, Zeiss) was used to collect the image.

### Decidua tissue staining

The decidua tissue was fixed with 4% paraformaldehyde, embedded in paraffin wax, and sectioned at a thickness of 5 μm. These sections underwent routine histological staining, and H&E staining was conducted to evaluate the tissue's morphological features. To assess the extent of fibrosis in the decidua, sections were processed with Masson's Trichrome staining.

## ELISA

The concentrations of CXCL1, IL‐6, IFN‐β, PRL, and IGFBP1 were quantified using ELISA kits. All assays were conducted following the manufacturer's instructions provided with the reagent kits. The optical density was measured at a wavelength of 450 nm using a microplate reader.

### SA‐β‐gal assay

T‐hESCs from different groups were cultured in 24‐well plates, followed by SA‐β‐gal staining to evaluate β‐galactosidase activity of cells. The samples were observed under a microscope (VS120‐S6‐W, Olympus) and analyzed using Image J (version 1.53 v, National Institutes of Health).

### Flow cytometry detection of cellular senescence

Cellular senescence was assessed using the SPiDER‐βGal Kit following the manufacturer's protocol. Cells from different groups were treated with Bafilomycin A1 for 1 h, then stained with SPiDER‐βGal working solution (1:1000 dilution, 30 min). After washing, cells were trypsinized, resuspended in culture medium, and analyzed by flow cytometry (excitation 488 nm; emission 515–545 nm) (Cytoflex S, Beckman Coulter, Brea, CA, USA). Data were analyzed with CytExpert 2.4.0.28.

### R‐loop detection using dot blot

The samples were isolated from mouse decidua tissues following a standard protocol. The sample's concentration was measured using a spectrophotometer. To prepare the control, a portion of the sample was treated under digestion conditions. Subsequently, the prepared samples were dabbed onto a nitrocellulose membrane. The membrane was air‐dried and exposed to UV cross‐linking. The membrane was blocked with 5% non‐fat dry milk in TBST for 1 h. The membrane was then incubated with the S9.6 antibody, followed by the corresponding secondary antibody. After washing, the signal was visualized using electrochemiluminescence substrate.

### Quantitative reverse transcription polymerase chain reaction (qRT‐PCR)

Total RNA was extracted from the cells using the FastPure Cell/Tissue Total RNA Isolation Kit V2. Following total RNA extraction, qRT‐PCR was performed using the HiScript II One Step qRT‐PCR SYBR Green Kit. Primers for the DHX9 are listed below: Forward primer (mouse): TATCCGAGGGGCTACTGGTTGT; Reverse primer (mouse): GCACTGATCCTTCTGGGCTGT; Forward primer (human): ATGACCCACTTTGTTCCTCCACC; Reverse primer (human): AGCCTCGATGAGTTCAAAAGGAGT.

### RNA‐seq experiment and high‐throughput sequencing

Total RNA extraction from T‐hESCs was performed using TRIzol reagent. For library preparation, the total RNA was used as input for RNA sequencing library preparation utilizing the KC^TM^ Digital mRNA Library Prep Kit, according to the manufacturer's instructions. The library preparation included the enrichment of PCR products corresponding to fragments ranging from 200 to 500 base pairs. The enriched libraries were quantified and sequenced on a DNBSEQ‐T7 platform (MGI) using the PE150 sequencing mode to generate paired‐end reads. Reads were aligned to the human reference genome Ensembl GRCh38 release 110 with STAR (version 2.7.6a). Reads mapping to exonic regions of each gene and to each transcript were counted separately using featureCounts (Subread‐1.5.1; Bioconductor), and then expression was calculated. Differentially expressed genes between groups were identified using the edgeR package (version 4.3.0), and a *p*‐value cutoff of 0.05 and |log_2_FC| ≥ 1 were used. Functional enrichment analysis (GO‐BP and KEGG) of the differentially expressed genes was performed using Metascape (https://metascape.org/) [62].

### Single‐cell RNA sequencing (scRNA‐seq)

Decidua tissue samples were minced into small fragments and subjected to enzymatic digestion in a thermomixer at 37°C for 1 h. Then, the red blood cells were separated, and the remaining cells were resuspended. Approximately 2 × 10^4^ cells were dispensed into each channel of the DPBio single‐cell chip (CRPS2A, DPBio), aiming to capture around 10^4^ target cells per channel. After encapsulation, cell lysis was performed, and the mRNAs were captured and barcoded using reverse transcription within the droplet. The cDNA was amplified, and the amplification product was evaluated to construct the sequencing library. Finally, sequencing was conducted on the MGI T7 platform (MGI), achieving a minimum depth of 20,000 reads per cell. For data analysis, dimensionality reduction was achieved through t‐SNE. Principal component analysis, cell clustering, and cell‐type annotation were performed using the Seurat R package (version 4.3.0). Additionally, the same package was utilized to perform the DEGs analysis. For each experimental group (control, URSA, hyperoside‐treated), three biological replicates were used for scRNA‐seq library preparation. Decidual tissues from each mouse were processed separately.

### Limited proteolysis mass spectrometry (Lip‐MS)

Cells were lysed in PBS. Following this, the lysates were equally distributed and incubated with either hyperoside at concentrations of 50 or 100 μM. Limited proteolysis was initiated by Proteinase K (PK) treatment, followed by reduction with dithiothreitol (DTT), alkylation with iodoacetamide (IAA), and trypsin digestion. Peptides were desalted, lyophilized, and reconstituted in 0.1% formic acid (FA) spiked with iRT standard peptides for retention time alignment. DIA‐MS analysis was conducted using a Vanquish Neo UHPLC system coupled with an Astral mass spectrometer (Thermo Scientific). Samples were analyzed in DIA mode with the following parameters: precursor ion scan range of 380–980 m/z, MS1 resolution of 2.4 × 10^5^ (at 200 m/z), normalized AGC target of 500%, and a maximum injection time of 5 ms. To maximize peptide ion coverage, the DIA acquisition was configured with 299 scan windows. The isolation window was fixed at 2 m/z, with HCD collision energy set to 25 eV. For MS2 analysis, the normalized AGC target and maximum injection time were set to 500% and 3 ms, respectively. DIA data were processed via Spectronaut software with a Q‐value cutoff of 0.01 (FDR < 1%). Differentially abundant proteins were defined by a fold change >1.5 or <0.67 and a *p*‐value < 0.05. To identify structural changes, we screened for peptides within the same protein sequence showing differential regulation, specifically targeting peptides that were up‐regulated in longer fragments but down‐regulated in shorter fragments, or those exhibiting bidirectional regulation in adjacent sequence segments. Finally, functional enrichment analyses (GO‐BP, KEGG) on differentially abundant proteins were performed using Metascape (https://metascape.org/) [62].

### Acquisition of cell senescence‐related genes and R‐loop regulators

Cell senescence‐related genes were retrieved from three publicly accessible databases: CellAge (https://genomics.senescence.info/cells/) [63], GenAge (https://genomics.senescence.info/genes/) [64], and GeneCards (https://www.genecards.org/) [65]. In addition, R‐loop regulators were collected from R‐loopBase (https://rloopbase.nju.edu.cn/) [66], a specialized database for R‐loop‐associated factors.

### Molecular docking model construction

The crystal structure of the DHX9 protein (PDB ID: 8SZP) was obtained from the Protein Data Bank (https://www.rcsb.org/) [67], and the structure of the compound (PubChem ID: 5281643) (https://pubchem.ncbi.nlm.nih.gov/) [68] was retrieved from the PubChem database. KVFinder was utilized to identify the potential binding pocket. Molecular docking between DHX9 and the compound was performed using AutoDock Vina. Binding interactions were analyzed with Discovery Studio Client, and docking conformations were visualized using PyMOL.

### Molecular dynamics (MD) simulations

MD simulations were performed using GROMACS 2022.3. Small molecule parameters were generated using AmberTools22, utilizing the GAFF force field for parameterization. Hydrogen atoms were added, and RESP charges were calculated using Gaussian16W. These parameters were compiled into the system's topology file. Following NaCl neutralization, the systems underwent energy minimization via the steepest descent method. Subsequently, the systems were subjected to equilibration processes, including isothermal isovolumic ensemble and isothermal isobaric ensemble, each consisting of 100,000 steps with a coupling time of 0.1 ps and a total duration of 100 ps. Finally, production runs were conducted under periodic boundary conditions for 100 ns at 26.85°C and 1.0 bar.

### Construction of DHX9 (T419A) mutant protein

To validate the key residues involved in the DHX9‐compound interaction, we expressed and purified the DHX9 (T419A) mutant protein. The main procedures are as follows: the amino acid sequence of DHX9 (T419A) from positions 325 to 840 was codon‐optimized and cloned into the pET28a vector. The N‐terminal sequence of DHX9 includes a 6×His tag and a SUMO tag. The DHX9 (T419A) expression vector was transformed into BL21‐CodonPlus‐RIPL expression strain. Following cultivation and induction, the protein was purified using a Ni column.

### SPR assay

SPR assay was performed using a Biacore 1K system to study the molecular interaction between hyperoside and DHX9. DHX9 (WT) or DHX9 mutant (T419A) protein was immobilized on a CM5 sensor chip (6000–7000 resonance units) using standard amine coupling chemistry and an amino coupling kit. Hyperoside in phosphate‐buffered saline Tween‐20 (PBST) was prepared at specified concentrations, with contact times of 60 s and dissociation times of 120 s. Data were acquired and analyzed using Biacore Insight Evaluation Software (version 5.0.18.22102) via a steady‐state 1:1 binding model or kinetic fitting.

## CETSA

Cells were treated with either hyperoside or DMSO for 1 h. Following treatment, the cells were lysed. To remove unlysed cell debris, the lysates were centrifuged at 20,000 *g* at 4°C. The resulting lysates were then divided into aliquots and subjected to heat treatment at a temperature gradient ranging from 37°C to 69°C (specifically: 37°C, 41°C, 45°C, 49°C, 53°C, 57°C, 61°C, 65°C, and 69°C) for 3 min at each temperature group. After heat treatment, the samples were cooled on ice for 3 min. Subsequently, the samples were centrifuged again at 20,000 *g* at 4°C to separate soluble proteins from denatured precipitates. The supernatants were collected for further analysis, and protein quantification was performed using Western blot assays.

### Statistical analysis

All data are presented as means ± SEM. Data analysis was performed using R (version 4.3.0) and GraphPad Prism 9.0. Statistical significance was set at *p* < 0.05, determined using two‐tailed Student's *t*‐tests or one‐way ANOVA with Tukey or Dunnett's post hoc tests. For comparisons between two groups, a two‐tailed Student's *t*‐test was used. For comparisons among three or more groups, one‐way ANOVA was performed, followed by: Tukey's post hoc test for all pairwise comparisons among groups; Dunnett's post hoc test for comparisons of each group against a single reference group.

## AUTHOR CONTRIBUTIONS

**Yuepeng Jiang**: Conceptualization; investigation; validation; methodology; writing—original draft. **Hongli Zhao**: Conceptualization; validation; methodology; software; writing—original draft. **Xinyi Ding**: Formal analysis; project administration; software; methodology; validation; investigation. **Haoling Zhang**: Conceptualization; investigation; validation; methodology; visualization; formal analysis; writing—original draft. **Yadong Guo**: Data curation; software; formal analysis; validation; investigation. **Yiming Ma**: Methodology; validation; investigation; conceptualization; formal analysis; software. **Lingyi Cai**: Conceptualization; investigation; visualization; project administration. **Qingnan Fan**: Software; validation; methodology; visualization. **Ruisi Peng**: Investigation; validation; visualization. **Fuyuan Yang**: Conceptualization; writing—original draft; formal analysis; visualization. **Doblin Sandai**: Conceptualization; formal analysis; project administration; validation; visualization; methodology. **Xianling Cao**: Methodology; validation; visualization; formal analysis. **Jiali Yao**: Software; formal analysis; data curation; resources; validation; visualization. **Wenyi Wang**: Validation; visualization; investigation; software; formal analysis. **Zhiheng Lin**: Methodology; validation; investigation; visualization. **Wangzheqi Zhang**: Methodology; validation; investigation; data curation. **Jialin He**: Conceptualization; writing—original draft; investigation; validation; methodology; formal analysis; software; project administration. **Aihua Zhang**: Software; formal analysis; validation; methodology; conceptualization; investigation. **Xiaoxuan Zhao**: Conceptualization; funding acquisition; writing—original draft; writing—review and editing; visualization; formal analysis; project administration; supervision.

## CONFLICT OF INTEREST STATEMENT

The authors declare no conflicts of interest.

## ETHICS STATEMENT

The project was conducted in compliance with the guidelines outlined in the Declaration of Helsinki and the Basel Declaration. Studies involving human tissues were approved by the Ethics Committee of Hangzhou Hospital of TCM affiliated to ZCMU (Hangzhou, China) (No. 2024KLL119). The study was performed in accordance with the principles of the Declaration of Helsinki. Written informed consent was obtained from all participants for the use of their clinical information. The animal experiments were approved by the Institutional Animal Care and Use Committee at Zhejiang Chinese Medical University (No. IACUC‐202505‐13).

## Supporting information


**Figure S1:** Chemical structure, extracted ion chromatogram, and MS/MS spectrum of hyperoside standard.
**Figure S2:** Hyperoside alleviates DNA damage, p53/Rb activation, and inflammatory cytokine expression in URSA models.
**Figure S3:** Measurement of cell viability, CXCL1, and IL‐6 after HYP treatment, along with the flow cytometric gating strategy used to identify SPiDER‐βGal^+^ cells, and the immunofluorescence intensity of γH2AX.
**Figure S4:** Overlaps of URSA DSC genes with hyperoside treatment and GO‐BP enrichment, and representative bar graphs showing quantitative analysis of S9.6, ssDNA, γH2AX, p53, and Rb expression from different experimental groups.
**Figure S5:** Comparative transcriptomics of RNase H1 overexpression in H_2_O_2_‐exposed T‐hESCs coupled with cGAS‐STING immunofluorescence validation in normal pregnancy versus URSA.
**Figure S6:** cGAS and STING expression in vimentin‐positive cells and IFN‐β levels in different groups.
**Figure S7:** Immunofluorescence for cGAS and STING among groups were measured.
**Figure S8:** GO‐BP and KEGG enrichment of hyperoside targets, molecular dynamics simulation of the hyperoside‐DHX9 complex, and SPR dynamic responses from hyperoside‐DHX9.
**Figure S9:** Western blot analysis of DHX9 expression and immunofluorescence intensity of γH2AX, p53, and Rb.
**Figure S10:** DHX9 knockdown abrogates the anti‐senescence effects of hyperoside in vivo.
**Figure S11:** Hyperoside exerts its anti‐senescence effects in mice by targeting the T421 residue of DHX9.
**Figure S12:** Identification of the most effective DHX9 shRNA by qRT‐PCR.

**Table S1:** R‐loop regulators.
**Table S2:** MM‐GBSA calculation results of DHX9‐HYP complex (mean ± SEM).
**Table S3:** Baseline characteristics of participating patients (Mean ± SD).
**Table S4:** Plasmid vectors and their sequence information (LV).
**Table S5:** Plasmid vectors and their sequence information (AAV).

## Data Availability

The raw sequence data reported in this paper have been deposited in the Genome Sequence Archive in the National Genomics Data Center, China National Center for Bioinformation/Beijing Institute of Genomics, Chinese Academy of Sciences (HRA018844, CRA043601, and OMIX017758), which are accessible at https://ngdc.cncb.ac.cn/gsa-human/browse/HRA018844, https://ngdc.cncb.ac.cn/gsa/browse/CRA043601, and https://ngdc.cncb.ac.cn/omix/release/OMIX017758. The human omics data generated in this study involve potentially sensitive information and are not publicly available due to ethical and privacy considerations. Access to the data may be granted upon reasonable request to the corresponding author, subject to approval by the relevant institutional review board and compliance with applicable data protection regulations. The data used are saved in GitHub: https://github.com/ZYZY156/Raw-data. Supplementary materials (figures, tables, graphical abstract, slides, videos, Chinese translated version, and updated materials) may be found in the online DOI or iMeta Science (http://www.imeta.science). The data that support the findings of this study are available from the corresponding author upon reasonable request.
