# Supplementary material for: Hyperoside alleviates endometrial stromal cell senescence in unexplained recurrent spontaneous abortion via DHX9‐mediated R‐loop resolution
Source: Imeta. 2026 Jul 13:e70150. Online ahead of print. doi: 10.1002/imt2.70150 (PMC13401611; doi:10.1002/imt2.70150)
Supplement: Supplementary file 1 — Figure S1: Chemical structure, extracted ion chromatogram, and MS/MS spectrum of hyperoside standard. Figure S2: Hyperoside alleviates DNA damage, p53/Rb activation, and inflammatory cytokine expression in URSA models. Figure S3: Measurement of cell viability, CXCL1, and IL‐6 after HYP treatment, along with the flow cytometric gating strategy used to identify SPiDER‐βGal+ cells, and the immunofluorescence intensity of γH2AX. Figure S4: Overlaps of URSA DSC genes with hyperoside treatment and GO‐BP enrichment, and representative bar graphs showing quantitative analysis of S9.6, ssDNA, γH2AX, p53, and Rb expression from different experimental groups. Figure S5: Comparative transcriptomics of RNase H1 overexpression in H2O2‐exposed T‐hESCs coupled with cGAS‐STING immunofluorescence validation in normal pregnancy versus URSA. Figure S6: cGAS and STING expression in vimentin‐positive cells and IFN‐β levels in different groups. Figure S7: Immunofluorescence for cGAS and STING among groups were measured. Figure S8: GO‐BP and KEGG enrichment of hyperoside targets, molecular dynamics simulation of the hyperoside‐DHX9 complex, and SPR dynamic responses from hyperoside‐DHX9. Figure S9: Western blot analysis of DHX9 expression and immunofluorescence intensity of γH2AX, p53, and Rb. Figure S10: DHX9 knockdown abrogates the anti‐senescence effects of hyperoside in vivo. Figure S11: Hyperoside exerts its anti‐senescence effects in mice by targeting the T421 residue of DHX9. Figure S12: Identification of the most effective DHX9 shRNA by qRT‐PCR. [file IMT2-9999-e70150-s002.doc]

**Supporting information to**

**Hyperoside alleviates endometrial stromal cell senescence in unexplained recurrent spontaneous abortion via DHX9-mediated R-loop resolution**

**Running title: Hyperoside targets DHX9 to resolve R-loop in URSA**

Yuepeng Jiang^1, 2#^, Hongli Zhao^1#^, Xinyi Ding^2#^, Haoling Zhang^3#^, Yadong Guo^4#^, Yiming Ma^5^, Lingyi Cai^6^, Qingnan Fan^6^, Ruisi Peng^6^, Fuyuan Yang^6^, Doblin Sandai^3^, Xianling Cao^7^, Jiali Yao^8^, Wenyi Wang^9^, Zhiheng Lin^10*^, Wangzheqi Zhang^11*^, Jialin He^1^*, Aihua Zhang^12^*, Xiaoxuan Zhao^1^*

^1^ Department of Traditional Chinese Medicine (TCM) Gynecology, Hangzhou TCM Hospital Affiliated to Zhejiang Chinese Medical University, Hangzhou 310007, China.

^2^College of Basic Medical Sciences, Zhejiang Chinese Medical University, Hangzhou 310053, China.

^3^ Department of Biomedical Sciences, Pusat Kanser Tun Abdullah Ahmad Badawi, University Sains Malaysia, Kepala Batas Pulau Pinang 13200, Malaysia.

^4^ Shanghai Tenth People’s Hospital, Tongji University. 301 Yanchang Road, Jing'an District, Shanghai 200040, China.

^5^ Faculty of Chinese Medicine, Macau University of Science and Technology, Macau 999078, China.

^6^ The Third Clinical Medical College, Zhejiang Chinese Medical University, Hangzhou 310053, China.

^7^ Harvard University Joslin diabetes center, Department of Medicine Harvard Medical School (HMS), Boston 02215, USA.

^8^Clinical Cancer Institute, Center for Translational Medicine, Naval Medical University, Shanghai 200433, China.

^9^ Juntendo University, 2-1-1 Hongo, Bunkyo-ku, Tokyo 113-8421, Japan.

^10^ Department of Gynecology, Longhua Hospital, Shanghai University of Traditional Chinese Medicine, Shanghai 200032, China.

^11^ Naval Medical University, Shanghai 200433, China.

^12^ Hainan Medical University, Haikou 571199, China.

^#^ These authors contributed equally: Yuepeng Jiang, Hongli Zhao, Xinyi Ding, Haoling Zhang, Yadong Guo

*Correspondence: [linzhiheng2020@163.com](mailto:linzhiheng2020@163.com) (Zhiheng Lin); [zwzq001031@smmu.edu.cn](mailto:zwzq001031@smmu.edu.cn) (Wangzheqi Zhang); [hejltcm@163.com](mailto:hejltcm@outlook.com) (Jialin He); [aihuatcm@163.com](mailto:aihuatcm@163.com) (Aihua Zhang); [tychexuan@163.com](mailto:tychexuan@163.com) (Xiaoxuan Zhao)

**Figure S1 Chemical structure, extracted ion chromatogram, and MS/MS spectrum of hyperoside standard.** (A) Chemical structure of hyperoside. (B) Extracted ion chromatogram (EIC) for hyperoside. (C) MS/MS spectrum of hyperoside standard.

**Figure S2 Hyperoside alleviates DNA damage, p53/Rb activation, and inflammatory cytokine expression in URSA models.** (A-C) IF co-staining was used to detect γH2AX (A), p53 (B), and Rb (C) in vimentin-positive cells of decidua tissue from control and URSA patients (n = 15). Scale bar: 50 μm. (D) Immunofluorescence (IF) co-staining was used to detect γPhospho-Histone H2AX (Ser139) (γH2AX). Scale bar: 25 μm. (E) IF co-staining was used to detect γH2AX, p53, and Rb in vimentin-positive cells from different groups (n = 3). (F) Western blot of γH2AX, p53, and Rb expression in decidua tissues from mice in the control, URSA, low-dose, medium-dose, high-dose hyperoside treatment, and dydrogesterone treatment groups. (G, H) ELISA assay was used to determine the level of CXCL1 and IL-6 from each group (n = 6). Data are presented as means ± SEM. Statistical significance is determined by two-tailed Student’s *t* test or one-way ANOVA (Dunnett’s post-test). ^*^*p* < 0.05 vs. control group; ^**^*p* < 0.01 vs. control group; ^#^*p* < 0.05 vs. URSA group.

**Figure S3 Measurement of cell viability, CXCL1, and IL-6 after HYP treatment, along with the flow cytometric gating strategy used to identify SPiDER-βGal⁺ cells, and the immunofluorescence intensity of γH2AX.** (A) CCK-8 assay was performed to measure the cell viability after HYP treatment in each group (n = 5). (B, C) ELISA assay was used to determine the level of CXCL1 (B) and IL-6 (C) from each group (n = 6). (D) Flow cytometric gating strategy for identification of SPiDER-βGal⁺ cells. FSC: Forward Scatter, SSC: Side Scatter. (E) The immunofluorescence intensity of γH2AX. Quantitative analysis and representative images of γH2AX (green), and nuclei (blue) are presented (n = 3). Scale bar: 10 μm. Data are presented as means ± SEM. Statistical significance is determined by one-way ANOVA (Dunnett’s post-test). ^*^*p* < 0.05 vs. HYP (0 μM) group; ^**^*p* < 0.01 vs. control group; ^#^*p* < 0.05 vs. H_2_O_2_ induction group; ^##^*p* < 0.01 vs. H_2_O_2_ induction group.

**Figure S4 Overlaps of URSA DSC genes with hyperoside treatment and GO-BP enrichment, and representative bar graphs showing quantitative analysis of S9.6, ssDNA, γH2AX, p53, and Rb expression from different experimental groups**. (A) Gene ontology biological process (GO-BP) enrichment analysis was performed on the overlapping gene set, defined as the intersection of genes upregulated in decidual stromal cells (DSCs) from URSA model and genes downregulated in DSCs after hyperoside treatment. (B) GO-BP enrichment analysis was performed on the overlapping gene set, defined as the intersection of genes downregulated in URSA DSCs and genes upregulated in DSCs following hyperoside treatment. (C) Quantitative analysis of S9.6 and ssDNA across experimental groups (n = 3). (D) Comparison of S9.6 and ssDNA levels among groups (n = 3). (E) Quantitative analysis of γH2AX, p53, Rb across experimental groups (n = 3). (F-H) IF staining was used to detect the immunofluorescence intensity of γH2AX (F), p53 (G), and Rb (H) among groups. Representative images of γH2AX (green), p53 (green), Rb (green) and nuclei (blue) are presented. Scale bar: 10 μm. Data are presented as means ± SEM. Statistical significance is determined by one-way ANOVA (Dunnett’s or Tukey post-test). **p* < 0.05 vs. vector control group; ***p* < 0.01 vs. control or con + vector control group; #*p* < 0.05 vs. H_2_O_2_ or H_2_O_2_ + vector control group; ##*p* < 0.01 vs. H_2_O_2_ + vector control group; &*p* < 0.05 vs. H_2_O_2_ + RNase H1 OE group.

**Figure S5 Comparative transcriptomics of RNase H1 overexpression in H₂O₂-exposed T-hESCs coupled with cGAS-STING immunofluorescence validation in normal pregnancy versus URSA**. (A) GO-BP enrichment analysis of the differentially expressed genes. (B) KEGG enrichment analysis of the differentially expressed genes. (C, D) IF co-staining was used to detect cGAS and STING in vimentin-positive cells from patients with normal pregnancy and URSA subjects (n = 15). Scale bar: 50 μm. Statistical significance is determined by two-tailed Student’s *t* test. ^**^*p* < 0.01 vs. Control group

**Figure S6 cGAS and STING expression in vimentin-positive cells and IFN-β levels in different groups**. (A, B) IF co-staining was used to detect cGAS (A) and STING (B) in vimentin-positive cells from mice with control, URSA, low-dose, medium-dose, high-dose hyperoside treatment, and dydrogesterone treatment (n = 3). Scale bar: 25 μm. (C) The levels of IFN-β in each group were measured by ELISA (n = 6). Data are presented as means ± SEM. Statistical significance is determined by one-way ANOVA (Dunnett’s post-test). **p* < 0.05 vs. control group; ***p* < 0.01 vs. control group; #*p* < 0.05 vs. URSA group; ##*p* < 0.01 vs. URSA group.

**Figure S7 Immunofluorescence for cGAS and STING among groups were measured.** (A, B) Immunofluorescence for cGAS (A) and STING (B) among groups were measured. Representative images of cGAS (green), STING (green), and nuclei (blue) are presented (n = 3). Scale bar: 10 μm. Data are presented as means ± SEM. Statistical significance is determined by one-way ANOVA (Tukey post-test). ***p* < 0.01 vs. con + vector control group; ##*p* < 0.01 vs. H_2_O_2_ + vector control group; &*p* < 0.05 vs. H_2_O_2_ + RNase H1 OE group.

**Figure S8 GO-BP and KEGG enrichment of hyperoside targets, molecular dynamics simulation of the hyperoside-DHX9 complex, and SPR dynamic responses from hyperoside-DHX9**. (A) GO-BP enrichment analysis of the overlapping genes (targets between senescence-associated genes and the genes encoding proteins identified in low-dose hyperoside-treated cells). (B) KEGG enrichment analysis of the overlapping genes. (C) GO-BP enrichment analysis of the overlapping genes (targets between senescence-associated genes and the genes encoding proteins identified in high-dose hyperoside-treated cell). (D) KEGG enrichment analysis of the overlapping genes. (E) The average number of hydrogen bonds between each ligand throughout 100 ns simulations. (F) Radius of gyration (Rg) graph of DHX9 docked with hyperoside from 0 to 100 ns time scale. (G) Rg boxplot of the hyperoside-DHX9 complex. (H-K) The SPR dynamic response resulting from the interaction of hyperoside-DHX9 (MUT): Steady state Affinity model fitting results, the x-axis represents sample concentration, while the y-axis represents binding signal (H, J), Surface-bound hyperoside-DHX9 (MUT) profile (I, K).

**Figure S9 Western blot analysis of DHX9 expression and immunofluorescence intensity of γH2AX, p53, and Rb.** (A) Representative immunoblots and quantification analysis of DHX9 expression in the control group, H_2_O_2_ group, and hyperoside treatment group (n = 3). (B-D) IF staining was used to detect the immunofluorescence intensity of γH2AX (B), p53 (C), and Rb (D) (n = 3). Scale bar: 20 μm. Data are presented as means ± SEM. Statistical significance is determined by one-way ANOVA (Dunnett’s post-test) or two-way ANOVA. ^**^*p* < 0.01; ns: Non-significant.

**Figure S10 DHX9 knockdown abrogates the anti-senescence effects of hyperoside in vivo**. (A, B) Immunofluorescence was used to detect DHX9 fluorescence intensity in each group. Scale bar: 25 μm. The results demonstrated that DHX9 shRNA (identified in Figure S12) significantly reduced DHX9 fluorescence within vimentin-positive cells of the decidual tissue. (C) IF co-staining was used to detect S9.6 in the vimentin-positive cells of decidua tissue from each group. Scale bar: 50 μm (left), 10 μm (right). (D) Quantification of S9.6, cGAS, and STING across experimental groups (n = 3). (E-J) IF co-staining was used to detect γH2AX (E, H), p53 (F, I), and Rb (G, J) in the vimentin-positive cells of decidua tissue from each group (n = 3). Scale bar: 25 μm. (K) ELISA assay was used to detect the level of CXCL1 and IL-6 from each group (n = 6). (L) Rhodamine phalloidin staining of the vimentin-positive cells in decidua tissue from each group. Scale bar: 25 μm. (M) ELISA assay was used to detect the level of PRL and IGFBP1 from each group (n = 6). (N) Embryo resorption in each group were recorded (n = 6). Data are presented as means ± SEM. Statistical significance is determined by one-way ANOVA (Tukey post-test). ^*^*p* < 0.05; ^**^*p* < 0.01; ns: Non-significant.

**Figure S11 Hyperoside exerts its anti-senescence effects in mice by targeting the T421 residue of DHX9**. (A) IF co-staining was used to detect S9.6 in the vimentin-positive cells of decidua tissue from each group. Scale bar: 50 μm (left), 10 μm (right). (B) Quantification of S9.6, cGAS, and STING across experimental groups (n = 3). (C-H) IF co-staining was used to detect γH2AX (C, F), p53 (D, G), and Rb (E, H) in the vimentin-positive cells of decidua tissue from each group (n = 3). Scale bar: 25 μm. (I) ELISA assay was used to detect the level of CXCL1 and IL-6 from each group (n = 6). Data are presented as means ± SEM. Statistical significance is determined by one-way ANOVA (Tukey post-test). ^*^*p* < 0.05; ^**^*p* < 0.01; ns: Non-significant.

**Figure S12 Identification of the most effective DHX9 shRNA by qRT-PCR.** (A) The effect of DHX9 shRNA (LV) was detected by qRT-PCR. In this study, shRNA1 exhibited the most significant effect on the down-regulation of DHX9 compared with other interference sequences. (B) The effect of DHX9 shRNA (AAV) was detected by qRT-PCR. In this study, shRNA2 exhibited the most significant effect on the down-regulation of DHX9 compared with other interference sequences. Data are presented as means ± SEM. Statistical significance is determined by one-way ANOVA (Dunnett’s post-test). ***p* < 0.01 vs. scramble shRNA.
